# Mitochondrial DNA mutations drive aerobic glycolysis to enhance checkpoint blockade response in melanoma

**DOI:** 10.1038/s43018-023-00721-w

**Published:** 2024-01-29

**Authors:** Mahnoor Mahmood, Eric Minwei Liu, Amy L. Shergold, Elisabetta Tolla, Jacqueline Tait-Mulder, Alejandro Huerta-Uribe, Engy Shokry, Alex L. Young, Sergio Lilla, Minsoo Kim, Tricia Park, Sonia Boscenco, Javier L. Manchon, Crístina Rodríguez-Antona, Rowan C. Walters, Roger J. Springett, James N. Blaza, Louise Mitchell, Karen Blyth, Sara Zanivan, David Sumpton, Edward W. Roberts, Ed Reznik, Payam A. Gammage

**Affiliations:** 1 Cancer Research UK Scotland Institute, Glasgow, UK; 2 Computational Oncology Service, Memorial Sloan Kettering Cancer Center, New York, NY USA; 3 Centro Nacional de Investigaciones Oncológicas (CNIO), Madrid, Spain; 4 Centro de Investigación Biomédica en Red de Enfermedades Raras CIBERER, Madrid, Spain; 5 Structural Biology Laboratory and York Biomedical Research Institute, Department of Chemistry, The University of York, York, UK; 6 School of Cancer Sciences, University of Glasgow, Glasgow, UK; 7 Marie-Josée and Henry R. Kravis Center for Molecular Oncology, Memorial Sloan Kettering Cancer Center, New York, NY USA; 8 Urology Service, Memorial Sloan Kettering Cancer Center, New York, NY USA

**Keywords:** Cancer genetics, Cancer microenvironment, Cancer metabolism, Cancer, Cancer immunotherapy

## Abstract

The mitochondrial genome (mtDNA) encodes essential machinery for oxidative phosphorylation and metabolic homeostasis. Tumor mtDNA is among the most somatically mutated regions of the cancer genome, but whether these mutations impact tumor biology is debated. We engineered truncating mutations of the mtDNA-encoded complex I gene, *Mt-Nd5*, into several murine models of melanoma. These mutations promoted a Warburg-like metabolic shift that reshaped tumor microenvironments in both mice and humans, consistently eliciting an anti-tumor immune response characterized by loss of resident neutrophils. Tumors bearing mtDNA mutations were sensitized to checkpoint blockade in a neutrophil-dependent manner, with induction of redox imbalance being sufficient to induce this effect in mtDNA wild-type tumors. Patient lesions bearing >50% mtDNA mutation heteroplasmy demonstrated a response rate to checkpoint blockade that was improved by ~2.5-fold over mtDNA wild-type cancer. These data nominate mtDNA mutations as functional regulators of cancer metabolism and tumor biology, with potential for therapeutic exploitation and treatment stratification.

## Abstract (web summary)

Gammage and colleagues find that mitochondrial DNA mutations induce alterations in redox metabolism, a remodeled tumor microenvironment characterized by a loss of neutrophils and consequently enhanced responses to immunotherapy in melanoma.

## Main

It has been known for several decades that >50% of cancers bear somatic mutations of mtDNA^1^. The impact of mtDNA mutations in the germline, the most common cause of inherited metabolic disease in humans^2^, is well established. However, the biological and clinical relevance of mtDNA mutations in cancer remains contentious^1^. Recent efforts have yielded evidence for the recurrence and selection of mtDNA mutations in cancer; however, the majority of variants observed somatically have not been detected in mitochondrial disease or previously studied in the germline^3,4^.

Hotspot truncating mutations in mitochondrial complex I genes are a common feature of several cancers, with truncating mutations in complex I (*MT-ND5* in particular) being over-represented compared with mutations in genes encoding respiratory complexes III, IV and V^3^. As complex I is a major site of NADH oxidation^5^, we reasoned that the proximal impact of complex I truncating mutations would be a loss of NADH:ubiquinone oxidoreductase activity, resulting in a redox imbalance with broad downstream impacts on cell metabolism. Previous studies have explored complex I function in tumor growth through the use of potent inhibitors that have severe impacts on respiratory chain function and cell metabolism in both malignant and non-malignant cells^6,7^. Here, we aimed to assess complex I dysfunction in a physiologically relevant, cancer-cell-specific manner by designing mitochondria-targeted base editors^8^ to induce premature stop codons within mouse *Mt-Nd5*, analogous to hotspot mutations found in the human *MT-ND5* gene in tumors^3^.

## Results

### mtDNA base editing to induce *Mt-Nd5* truncating mutations

**Fig. 1:**
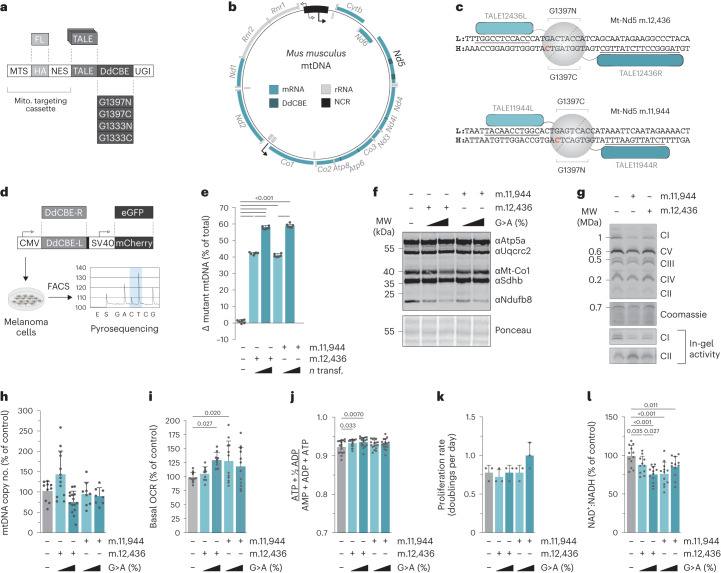
Mitochondrial base editing results in isogenic cell lines bearing two independent truncating mutations in *Mt-Nd5*. **a**, Schematic of the TALE-DdCBE design used. TALEs were incorporated into a backbone containing a mitochondria-targeting cassette, split-half DdCBE and uracil glycosylase inhibitor (UGI). MTS, mitochondrial targeting sequence; NES, nuclear export signal. **b**, Schematic of the murine mtDNA. Targeted sites within *Mt-Nd5* are indicated. **c**, TALE-DdCBE pairs used to induce a G>A mutation at m.12,436 and m.11,944. **d**, Workflow used to produce *Mt-Nd5* mutant isogenic cell lines. **e**, Heteroplasmy measurements of cells generated in **d** (*n* = 6 biological replicates). **f**, Immunoblot of indicative respiratory chain subunits. Representative result of three biological replicates is shown. **g**, Assembled complex I abundance and in-gel activity. Representative result of three biological replicates is shown. **h**, mtDNA copy number (*n* = 10, 13, 9, 15 and 8 technical replicates over *n* = 4, 5, 3, 5 and 3 biological replicates). **i**, Basal oxygen consumption rate (OCR) (*n* = 12, 9, 12, 9 and 12 technical replicates over *n* = 4, 3, 4, 3 and 4 biological replicates). **j**, Energy (adenylate) charge state (*n* = 17 technical replicates over *n* = 6 biological replicates). **k**, Proliferation rate of cell lines in permissive growth media. (*n* = 3 biological replicates). **l**, NAD^+^:NADH ratio (*n* = 12, 11, 12, 12 and 12 technical replicates over *n* = 4 biological replicates). *P* values were determined using a one-way ANOVA test with Sidak multiple comparisons test (**e**, **h**–**i**, **k**) or Fisher’s LSD test (**j**,**l**). Measure of centrality, mean; error bars, s.d. Number of replicates are described across conditions from left to right as presented. Source data

**Extended Data Fig. 1:**
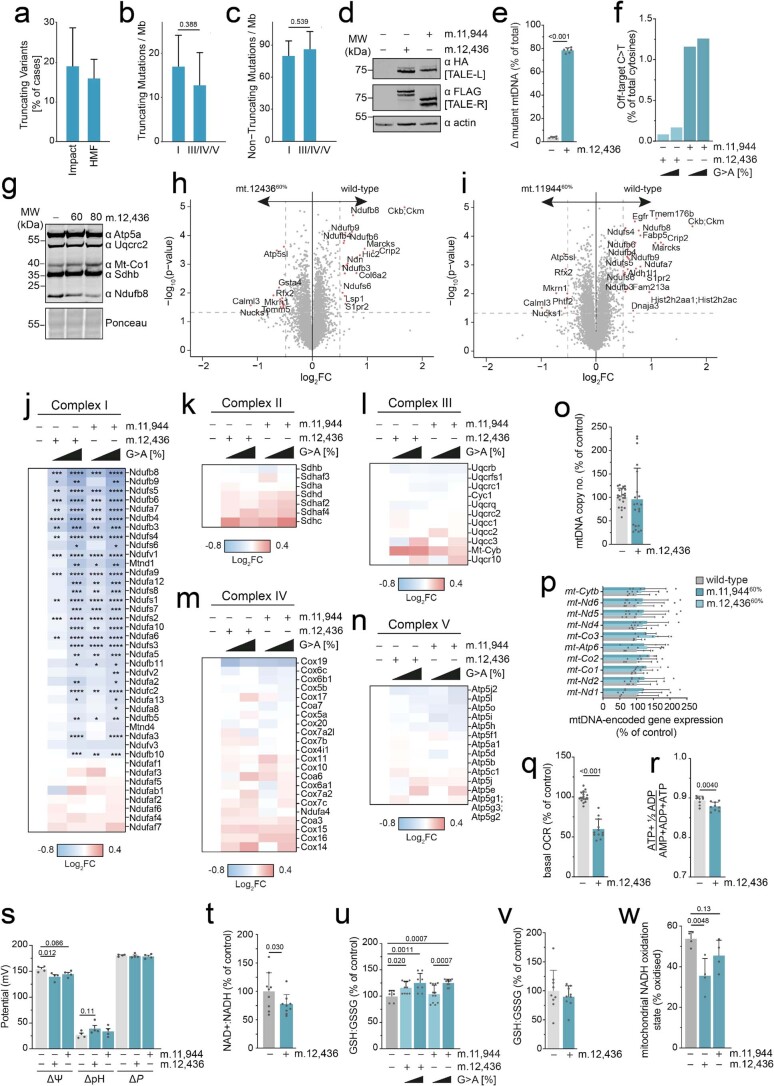
Characterising complex I truncating mutations in melanoma. **a** Unique truncating variants were detected in an average of 16% of melanoma patients (n = 281) and in 19% of melanoma patients (n = 89) from the Hartwig Medical Foundation (HMF) and MSK IMPACT melanoma cohorts respectively. **b** Truncating and **c** non-truncating mutations in melanoma patients, combined from both IMPACT and HMF patient cohorts, show that truncations are enriched in complex I, compared to complexes III, IV, and V. **d** Immunoblot of DdCBE pair expression post-sort. αHA and αFLAG show expression of left (TALE-L) and right TALEs (TALE-R) respectively. One biological replicate performed. **e** Heteroplasmy measurements of cells generated (n = 6 biological replicates). **f** Off-target C>T activity of DdCBEs on mtDNA. Figure shows mutations detected at heteroplasmies >2% and is a measure of mutations detected relative to wild-type. These mutations likely do not impact our key observations as both models behave similarly across experiments. **g** Immunoblot of indicative respiratory chain subunits. Representative result of three biological replicates is shown. Volcano plot showing detected differences in protein abundance of wild-type versus **h** mt.12436^60%^ cells and **i** mt.11944^60%^ cells. Differences of p-value < 0.05 and log_2_ fold change > 0.5 shown in red (n = 3 biological replicates). Heatmaps of protein abundances for **j** complex I, **k** complex II, **l** complex III, **m** complex IV and **n** complex V nuclear and mtDNA-encoded subunits (n = 3 biological replicates). **o** mtDNA copy number (n = 22 and 21 technical replicates over n = 8 and 7 biological replicates). **p** Expression of mitochondrial genes (n = 4 biological replicates). **q** Basal oxygen consumption rate (OCR) (n = 18 and 12 technical replicates over n = 6 and 4 biological replicates) **r** Energy (adenylate) charge state (n = 9 technical replicates over n = 3 biological replicates). **s** Measurements of the electrical component of the proton motive force, ΔΨ, the chemical component of the proton motive force ΔpH and total protonmotive force, ΔP (n = 4 biological replicates). **t** NAD+:NADH ratio (n = 9 technical replicates over n = 3 biological replicates). **u** GSH:GSSG ratio (n = 6, 10, 12, 8 and 10 technical replicates over n = 2, 4, 4 and 3 biological replicates). A high GSH:GSSG ratio represents a more reductive intracellular environment. **v** GSH:GSSG ratio (n = 9 technical replicates over n = 3 biological replicates) **w** Mitochondrial NADH oxidation state (n = 4 biological replicates). P-values were determined using a two-tailed Fisher’s exact test (A-C), student’s one-tailed t-test (E, O, Q, R, T, V), two-tailed Wilcoxon signed rank test (H, I), one-way ANOVA test with Sidak multiple comparisons test (J-N) or one-way ANOVA test with Fisher’s LSD test (S, U, W). Error bars indicate CI (A-C) or SD (E,O-W). Measure of centrality is mean. * P = < 0.05, ** P = <0.01, *** P = <0.001, **** P = <0.0001. Number of replicates are described across conditions from left to right as presented. Heatmap representations of data where asterisks are not present report non-significant changes. Source data

Between 16 and 19% of melanomas bear truncating mutations in complex I genes (Extended Data Fig. 1a–c). As such, transcription-activator-like effector (TALE)-DddA-derived cytosine base editor (DdCBE) G1397/G1333 candidates bearing nuclear export signals targeting m.12,436G>A and m.11,944G>A sites in Mt-Nd5 were synthesized and screened in mouse B78-D14 amelanotic melanoma cells (B.16 derivative, Cdkn2a^−/−^)^9^ to identify efficient pairs (Fig. 1a–d). Expression of lead pairs (Extended Data Fig. 1d) resulted in isogenic cell populations bearing ~40%, ~60% or ~80% mutation heteroplasmy of m.12,436G>A or ~40% or ~60% for m.11,944G>A truncating mutations following either a single transfection or up to four consecutive transfections (referred to as m.12,436^40%^, m.12,436^60%^, m.12436^80%^, m.11,944^40%^ and m.11,944^60%^, respectively) (Fig. 1e and Extended Data Fig. 1e) with a limited off-target mutation profile (Extended Data Fig. 1f). The resulting stable isogenic cell lines demonstrated a heteroplasmy-dependent decrease in expression of complex I subunit Ndufb8 without a substantial impact on other respiratory chain subunits (Fig. 1f and Extended Data Fig. 1g). This was supported by proteomic data (Extended Data Fig. 1h–n) and blue-native PAGE analysis of the m.12,436^60%^ and m.11,944^60%^ cell lines (Fig. 1g), confirming that individual complex I subunit abundance, in addition to the proportion of fully assembled complex I, is decreased in proportion to the mutant load without a substantial impact on other components of the oxidative phosphorylation system. In-gel activity assays of complex I and complex II activity further support this finding (Fig. 1g). mtDNA copy number was not impacted by mutation incidence or heteroplasmy level (Fig. 1h and Extended Data Fig. 1o), and the *Mt-Nd5* transcript level was unchanged in m.12,436^60%^ and m.11,944^60%^ mutant cells compared with controls, consistent with a lack of nonsense-mediated decay in mammalian mitochondria (Extended Data Fig. 1p). A significant decrease in oxygen consumption was detected only in m.12,436^80%^ cells (Fig. 1i and Extended Data Fig. 1q) without substantively impacting the adenylate energy charge state (Fig. 1j and Extended Data Fig. 1r) or cell proliferation (Fig. 1k). An ~10 mV decrease in the electrical component of the mitochondrial proton motive force, ΔΨ, coupled to a commensurate trend towards ~10 mV increases in the chemical component, ΔpH, resulting in an unchanged total proton motive force, Δ*P*, was detected in m.12,436^60%^ and m.11,944^60%^ mutant cells (Extended Data Fig. 1s) The NAD^+^:NADH ratio was significantly impacted in mutant cells (Fig. 1l and Extended Data Fig. 1t), which was also reflected in reduced:oxidized glutathione ratios (Extended Data Fig. 1u,v). The effect on cellular redox poise was further determined in m.12,436^60%^ and m.11,944^60%^ cells using NAD(P)H fluorescence (Extended Data Fig. 1w). Taken together, these data demonstrate that truncating mutations in *Mt-Nd5* exert heteroplasmy-dependent effects on the assembly of complex I. In turn, partial loss of complex I disrupts cellular redox balance without substantially impacting cellular energy homeostasis, gene expression or cell proliferation.

### Redox imbalance underpins a shift towards aerobic glycolysis

**Fig. 2:**
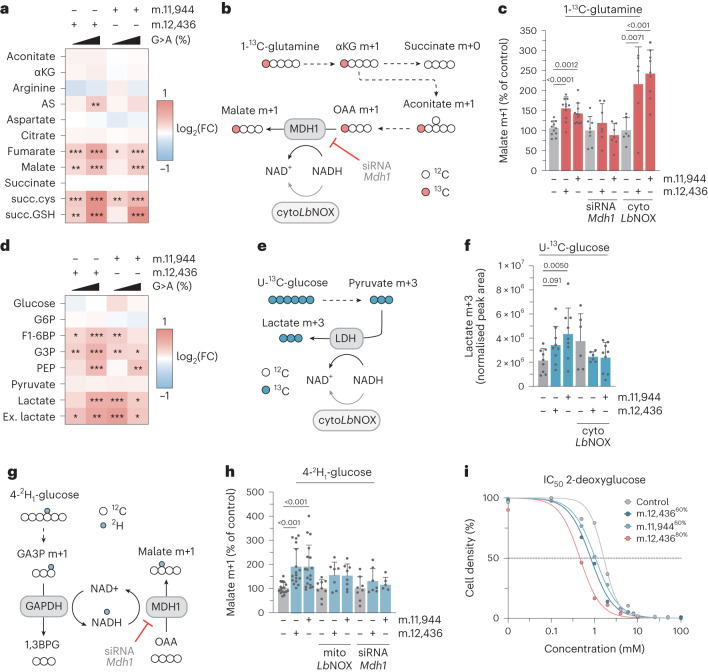
Mutant cells undergo a metabolic shift towards glycolysis caused by cellular redox imbalance. **a**, Heatmap of unlabeled steady-state abundance of select mitochondrial metabolites, arginine, argininosuccinate (AS) and terminal fumarate adducts succinylcysteine (succ.cys) and succinicGSH (succGSH) (*n* = 12–18 technical replicates over *n* = 6 biological replicates). FC, fold change. **b**, Labeling fate of ^13^C derived from 1-^13^C-glutamine. **c**, Malate m+1 abundance, derived from 1-^13^C-glutamine with indicated treatment (*n* = 11, 11, 11, 8, 8, 8, 6, 6 and 9 technical replicates over *n* = 4, 4, 4, 3, 3, 3, 3, 3 and 3 biological replicates). **d**, Heatmap of unlabeled steady-state metabolite abundances for select intracellular glycolytic intermediates and extracellular lactate (Ex. lactate) (*n* = 12–18 technical replicates over *n* = 6 biological replicates). **e**, Labeling fate of U-^13^C-glucose. **f**, Abundance of U-^13^C-glucose derived lactate m+3 with indicated treatment (*n* = 9, 9, 9, 9, 6 and 6 technical replicates over *n* = 3 biological replicates). **g**, Labeling fate of ^2^H derived from 4-^2^H_1_-glucose; mito*Lb*NOX not shown for clarity. **h**, Malate m+1 abundance, derived from 4-^2^H_1_-glucose with indicated treatment (*n* = 17, 17, 18, 9, 7, 8, 8, 7 and 5 technical replicates over *n* = 6, 6, 6, 3, 3, 3, 3, 3 and 2 biological replicates). **i**, IC_50_ curves for 2-deoxyglucose (*n* = 4 technical replicates). Representative result of three biological replicates is shown. *P* values were determined using a one-way ANOVA test with Sidak multiple comparisons test (**a**,**d**) or Fisher’s LSD Test (**c**,**f**,**h**). Measure of centrality, mean; error bars, s.d. *P < 0.05; **P < 0.01; ***P < 0.001. Number of replicates are described across conditions from left to right as presented. Heatmap representations of data for which asterisks are not present report non-significant changes. Source data

**Extended Data Fig. 2:**
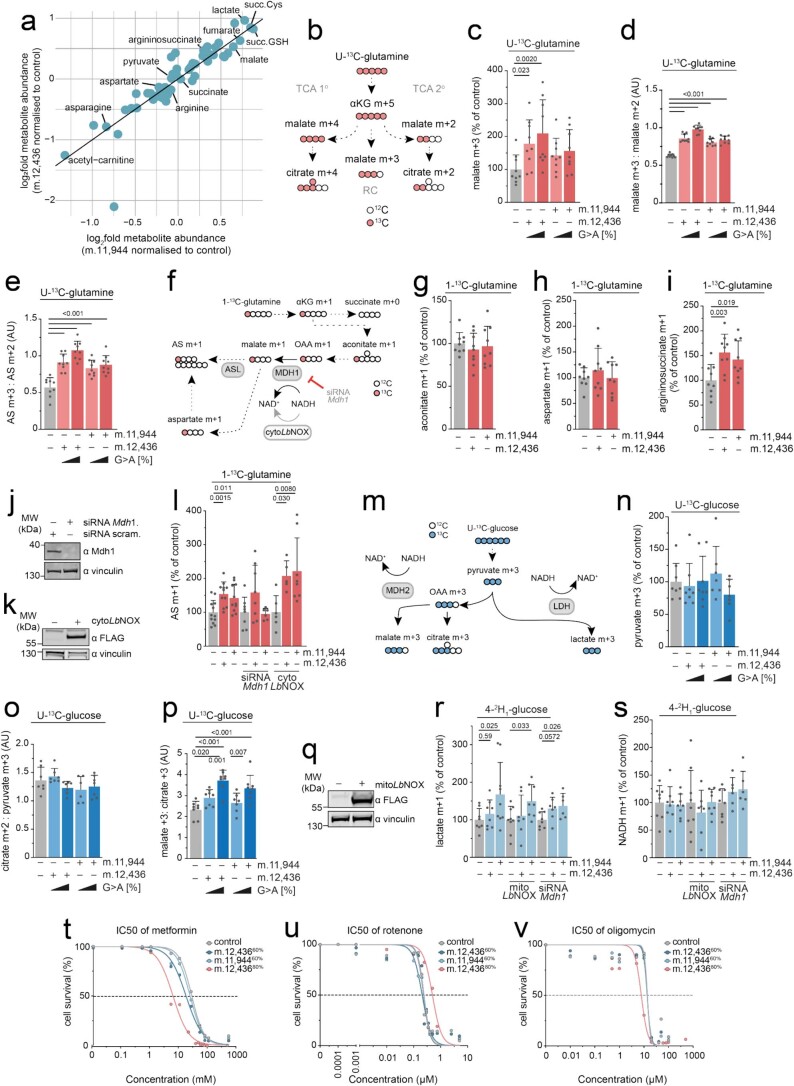
Independent mt-Nd5 truncations produce consistent changes in metabolic profile. **a** Comparison of steady-state metabolite changes of m.12,436^60%^ and m.11,944^60%^ cells, each relative to wild-type (n= 6–9 separate wells per genotype). **b** Labelling fate of ^13^C derived from U-^13^C-glutamine via oxidative decarboxylation versus reductive carboxylation of glutamine. **c** Malate m+3 abundance, derived from U-^13^C-glutamine (n = 9 technical replicates over n = 3 biological replicates). **d** malate m+3:malate m+2 ratio, derived from U-^13^C-glutamine (n = 9 technical replicates over n = 3 biological replicates). **e** AS m+3:AS m+2 ratio, derived from U-^13^C-glutamine (n = 9 technical replicates over n = 3 biological replicates). **f** Labelling fate of ^13^C derived from 1-^13^C-glutamine which exclusively labels metabolites derived from the reductive carboxylation of glutamine. **g** Aconitate m+1 abundance, derived from 1-^13^C-glutamine (n = 9 technical replicates over n = 3 biological replicates). **h** Aspartate m+1 abundance, derived from 1-^13^C-glutamine (n = 9 technical replicates over n = 3 biological replicates). **i** AS m+1 abundance, derived from 1-^13^C-glutamine (n = 9 technical replicates over n = 3 biological replicates). **j** Immunoblot of siRNA mediated depletion of *Mdh1*. Representative result of three biological replicates is shown. **k** Immunoblot of cyto*Lb*NOX expression 36hrs post-sort, detected using αFLAG. Representative result of three biological replicates is shown. **l** AS m+1 abundance, derived from 1-^13^C-glutamine with indicated treatment (n = 12, 11, 11, 8, 8, 6, 6, 5 and 8 technical replicates over n = 4, 4, 4, 3, 3, 2, 2, 2 and 3 biological replicates). **m** Labelling fate of ^13^C derived from U-^13^C-glucose. **n** Pyruvate m+3 abundance, derived from U-^13^C-glucose (n = 8, 8, 7, 8 and 7 technical replicates over n = 3 biological replicates). **o** Citrate m+2:pyruvate m+3 ratio, derived from U-^13^C-glucose (n = 7, 7, 6, 7 and 6 technical replicates over n = 3, 3, 2, 3 and 2 biological replicates). **p** Malate m+3:citrate m+3 ratio, derived from U-^13^C-glucose (n = 8, 8, 7, 8 and 7 technical replicates over n = 3 biological replicates). **q** Immunoblot of mito*Lb*NOX expression in sorted cells 36hrs post-transfection, detected using αFLAG. Representative result of three biological replicates is shown. **r** Lactate m+1 abundance, derived from 4-^2^H_1_-glucose with indicated treatment (n = 8, 8, 9, 9, 7, 8, 8, 7 and 6 technical replicates over n = 3, 3, 3, 3, 3, 3, 3, 3 and 2 biological replicates). **s** NADH m+1 abundance, derived from 4-^2^H_1_-glucose with indicated treatment (n = 8, 8, 8, 9, 7, 8, 8, 6 and 6 technical replicates over n = 3, 3, 3, 3, 3, 3, 3, 2 and 2 biological replicates). **t** IC_50_ curve for metformin. IC_50_ for wild-type = 26.31 ± 1.49mM, for mt.12436^60%^ = 16.60 ± 2.43mM, for mt.12436^80%^ = 5.89 ± 0.71mM and for mt.11944^80%^ = 22.93 ± 0.70mM **U** IC_50_ curve for rotenone. IC_50_ for wild-type = 0.236 ± 0.026µM, for mt.12436^60%^ = 0.235 ± 0.035µM, for mt.12436^80%^ = 0.493 ± 0.108µM and for mt.11944^60%^ = 0.205 ± 0.033µM and **V** IC_50_ curve for oligomycin. IC_50_ for wild-type = 13.81 ± 3.80µM, for mt.12436^60%^ = 13.52 ± 3.32µM, for mt.12436^80%^ = 7.75 ± 0.56µM and for mt.11944^80%^ = 13.54 ± 3.32µM (n = 4 technical replicates). Representative result of three biological replicates is shown. All P-values were determined using a one-way ANOVA test with Fisher’s LSD test. Error bars indicate SD. Measure of centrality is mean. Number of replicates are described across conditions from left to right as presented. Source data

Unlabeled metabolomic measurements from m.12,436^60%^ and m.11,944^60%^ cells revealed consistent differences in metabolite abundance in these cells relative to controls (Extended Data Fig. 2a), with notable increases in the steady-state abundance of malate, lactate, fumarate, argininosuccinate and the metabolically terminal fumarate adducts succinylcysteine and succinic glutathione (succinicGSH) (Fig. 2a). Heteroplasmy-dependent increases in the abundance of lactate and malate in the context of constant succinate in mutant cells suggested that the flow of electrons into mitochondria through the malate–aspartate shuttle (MAS) might be impacted by changes in the redox state of the cell. To study this possibility, we first measured the contributions of glutamine-derived carbon to tricarboxylic acid (TCA) cycle metabolites using U-^13^C-glutamine isotope tracing (Extended Data Fig. 2b). This indicated increased abundance of malate from cytosolic oxaloacetate, derived from citrate through ATP citrate lyase, as determined by the abundance of malate m+3 and the ratio of malate m+3:m+2, which demonstrated a significant, heteroplasmy-dependent increase relative to controls (Extended Data Fig. 2c,d), with a similar pattern of m+3:m+2 labeling observed for the cytosolic urea cycle metabolite argininosuccinate (Extended Data Fig. 2e). We then traced the metabolic fate of carbon from 1-^13^C-glutamine, which labels metabolites derived from the reductive carboxylation of glutamine (Fig. 2b and Extended Data Fig. 2f). This revealed that the increased abundance of malate m+1 occurred at the level of MDH1 (Fig. 2c) and was not apparent in downstream or upstream metabolites aconitate and aspartate (Extended Data Fig. 2g,h), with the m+1 labeling pattern of argininosuccinate again matching that of malate (Extended Data Fig. 2i). The increased abundance of malate m+1 and argininosuccinate AS+1 was sensitive to small interfering RNA (siRNA)-mediated depletion of *Mdh1* but was not diminished by expression of cytosolically targeted *Lb*NOX (cyto*Lb*NOX), a water-forming NADH oxidase^10^ (Fig. 2c and Extended Data Fig. 2j–l), indicating that increases in malate abundance occur at least partially in the cytosol through MDH1 but are not a result of gross alteration in cytosolic redox poise.

Elevated cellular and extracellular lactate, alongside the increased abundance of several glycolytic intermediates (Fig. 2d) suggested the use of pyruvate as an electron acceptor to rebalance NAD^+^:NADH through lactate dehydrogenase. Using U-^13^C-glucose tracing (Fig. 2e and Extended Data Fig. 2m), we observed increased abundance of lactate m+3 in m.12,436^60%^ and m.11,944^60%^ cells that was abolished by cyto*Lb*NOX expression (Fig. 2f). The increase in lactate m+3 was not accompanied by changes in pyruvate m+3 levels (Extended Data Fig. 2n) or by the entry of glucose-derived carbon into the TCA cycle through pyruvate dehydrogenase, determined by the ratio of citrate m+2:pyruvate m+3 (Extended Data Fig. 2o). However, the fate of carbon entering the TCA cycle through pyruvate carboxylase was substantially altered, with a malate m+3:citrate m+3 ratio indicative of MDH2 reversal (Extended Data Fig. 2p). Coupling of the MAS with glycolysis is a topic of recent interest, with several reports linking mitochondrial dysfunction with NADH shuttling between GAPDH and MDH1 or lactate dehydrogenase^11,12^. Using 4-^2^H_1_-glucose isotope tracing (Fig. 2g) we observed an increase in the abundance of malate m+1 in m.12,436^60%^ and m.11,944^60%^ cells, with a similar trend in lactate m+1 abundance, that was sensitive to expression of a mitochondrially targeted *Lb*NOX (mito*Lb*NOX) and siRNA-mediated depletion of *Mdh1* (Fig. 2h and Extended Data Fig. 2q–s), supporting the notion that the NAD^+^:NADH imbalance resulting from partial loss of complex I supports enhanced glycolytic flux by coupling cytosolic elements of the MAS with glycolysis. In turn, this increased dependence on glycolysis rendered m.12,436^60%^ (half-maximum inhibitory concentration, IC_50_ = 0.81 ± 0.064 mM) and m.11,944^60%^ cells (IC_50_ = 1.04 ±0.040 mM) more sensitive to the competitive phosphoglucoisomerase inhibitor 2-deoxyglucose compared with wild-type cells (IC_50_ = 1.62 ± 0.063 mM) (Fig. 2i), a sensitivity that was further enhanced in m.12,436^80%^ cells (IC_50_ = 0.46 ± 0.080 mM). The m.12,436^60%^ and m.12,436^80%^ cells demonstrated sensitivity to the low-affinity complex I inhibitor metformin relative to wild-type cells (Extended Data Fig. 2t). The ~60% mutants were not differentially sensitive to the potent complex I inhibitor rotenone, although m.12,436^80%^ cells demonstrated resistance compared to wild-type cells (Extended Data Fig. 2u). None of the mutants demonstrated differential sensitivity to the complex V inhibitor oligomycin (Extended Data Fig. 2v). Taken together, these data suggest that truncating mutations in *Mt-Nd5* of complex I induce a Warburg-like metabolic state through redox imbalance, not energetic crisis. This influences both cytosolic and mitochondrial components of the MAS, enhancing glycolytic flux and increasing sensitivity to the inhibition of this adaptive metabolic strategy as well as producing elevated levels of characteristic terminal fumarate adducts succinicGSH and succinylcysteine.

### mtDNA mutations reshape the tumor immune landscape

**Fig. 3:**
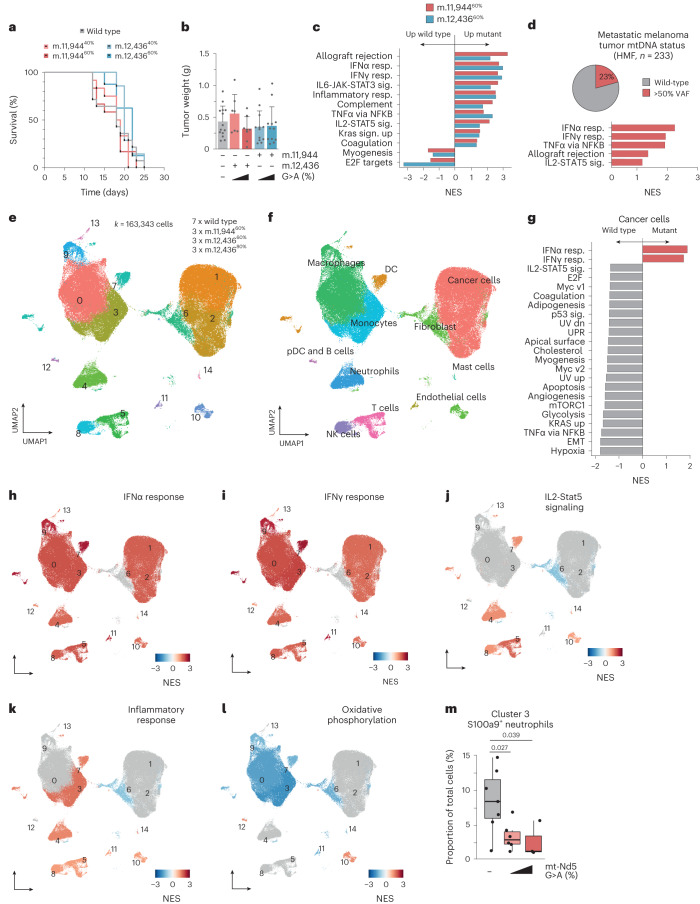
Tumor mtDNA mutations reshape the immune microenvironment. **a**, Survival of C57/BL6 mice subcutaneously injected with indicated cells (*n* = 14, 12, 7, 12 and 8 animals). **b**, Mean tumor weight at endpoint (*n* = 14, 12, 7, 12 and 8 individual tumors). Error bars, s.d. **c**, GSEA of bulk tumor RNA-seq data (*n* = 5–6 individual tumors per genotype). Only gene sets with *P*_adj_ < 0.1 are shown. TNFα, tumor necrosis factor alpha. **d**, GSEA of RNA-seq obtained from HMF database of patients with metastatic melanoma. Cancers are stratified by mtDNA status into wild type and mtDNA mutant with >50% VAF. **e**, UMAP of Seurat clustered whole tumor scRNA-seq from indicated samples. **f**, UMAP indicating cell type IDs. DC, dendritic cells; pDC, plasmacytoid dendritic cell; NK, natural killer. **g**, GSEA of malignant cells identified in scRNA-seq analysis. Comparison is wild-type tumors versus all mutant tumors. **h**–**l**, UMAPs colored by GSEA score for IFNα response (**h**), IFNγ response (**i**), IL2-Stat5 signaling (**j**), inflammatory response (**k**) and oxidative phosphorylation (**l**). **m**, Proportion of tumor-resident S100a9^+^ neutrophils relative to total malignant and non-malignant cells (*n* = 7, 6 and 3 individual tumors). Boxplots indicate mean and interquartile range; error bars, s.e.m. One-way ANOVA test with Sidak multiple comparisons test (**b**), two-tailed Wilcoxon signed rank test (**c**,**d**,**g**–**l**) and two-tailed Student’s *t*-test (**m**) were applied. Number of replicates are described across conditions from left to right as presented. Source data

**Extended Data Fig. 3:**
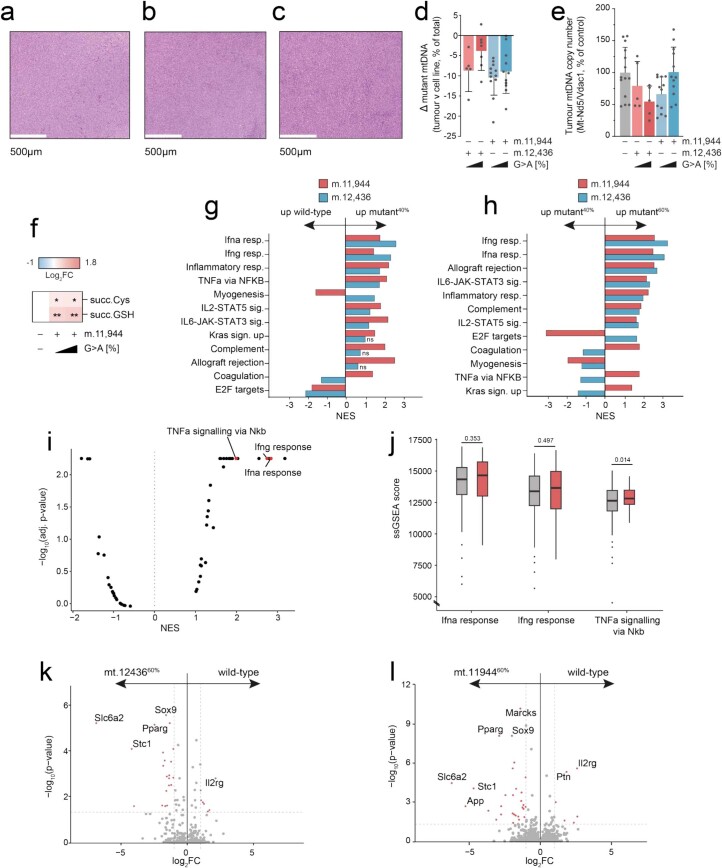
Characterisation of mtDNA mutant tumors in mice and humans. Representative H&E from 5 sub-sections of **a** wild-type, **b** m.12,436^40%^ and **C** m.12,436^60%^ tumors. **d** Change in detected heteroplasmy in bulk tumor samples (n = 5, 7, 12 and 11 individual tumours). **e** Bulk tumor mtDNA copy number (n = 14, 5, 5, 12 and 12 individual tumours). **f** Heatmap of steady-state abundance of metabolically terminal fumarate adducts, succinylcysteine and succinicGSH, demonstrating that metabolic changes observed *in vitro* are preserved *in vivo* (n = 9–11 individual tumours). GSEA of bulk tumor RNAseq data (n = 5-6 individual tumours) showing **g** mutant^40%^ versus wild-type and **h** mutant^60%^ versus mutant^40%^. **i** Volcano plot depicting NES and -log_10_(adj. p-value) derived from GSEA comparing mtDNA mutant >50% VAF to wild-type skin cancer samples from HMF. Pathways associated with the innate immune response are highlighted. **j** ssGSEA analysis comparing mtDNA mutant to wildtype skin cancer samples from HMF. Effect sizes are consistent with GSEA analysis, but do not reach statistical significance. Only genesets with adj. p-value <0.1 are shown unless otherwise stated. Grey, mtDNA wildtype (n = 256); red, >50% VAF (n = 57). Volcano plots showing differences in gene expression of wild-type versus **k** mt.12436^60%^ cells and **L** mt.11944^60%^ cells. Differences of p-adj < 0.05 and log_2_ fold change > 1 shown in red (n = 4 biological replicates). Wilcoxon signed rank test applied. A one-way ANOVA test with Fisher’s LSD test (F), two-tailed Wilcoxon signed rank test (I, K-L) or two-tailed student’s t-test were applied. Error bars indicate SD. Measure of centrality is mean. * P = < 0.05, ** P = <0.01. Number of replicates are described across conditions from left to right as presented. NES: normalised enrichment score. Heatmap representations of data where asterisks are not present report non-significant changes GSEA: gene-set enrichment analysis. SSGSEA: Single Sample GSEA. HMF: Hartwig Medical Foundation. Source data

**Extended Data Fig. 4:**
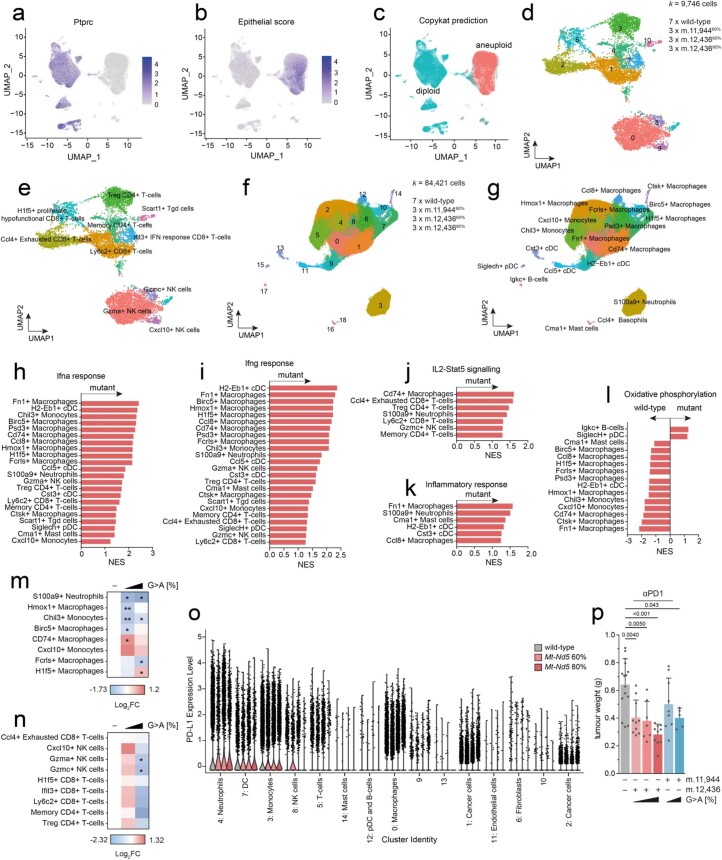
scRNAseq analyses reveal distinct alterations in the tumor immune microenvironment of mtDNA mutant tumors. UMAP indicating **a** Ptprc expression, **b** epithelial score and **c** aneuploidy as determined by copykat prediction. These criteria were employed as the B78 cells lack distinct transcriptional signatures to allow unambiguous identification of malignant cells. **d** UMAP of seurat clustered T-cell/ NK cell scRNAseq from indicated samples. **e** UMAP indicating cell type IDs. NK, natural killer cells. IFN, interferon. Tgd, gamma delta T-cells. Treg, regulatory T-cells. **f** UMAP of seurat clustered myeloid scRNAseq from indicated samples. **g** UMAP indicating cell type IDs. cDC, conventional dendritic cells. Malignant cells were identified for scRNAseq analysis as aneuploid cells with low or nil Ptprc (CD45) expression and high epithelial score. GSEA of non-malignant cells with significant changes for **h** Ifna response, **i** Ifng response, **j** IL2-STAT5 signaling, **k** inflammatory response and **l** oxidative phosphorylation. **m** Heatmap of tumor resident myeloid cells with significant changes (p < 0.1) in proportion relative to the total malignant and non-malignant cells (n = 7, 3, 3, and 3 individual tumours). **n** Heatmap of tumor resident lymphoid cells with significant changes (p < 0.1) in proportion relative to the total malignant and non-malignant cells (n = 7, 3, 3, and 3 individual tumours). **o** Relative PD-L1 expression within each cell cells (n = 7, 3, 3, and 3 individual tumours). 60% mutant tumors are coloured the same, presented as m.12,436G>A and m.11,944G>A from left to right. **p** Harvested tumor weight at day 21 (n = 15, 10, 7, 10, 9 and 5 individual tumours). Two-tailed Wilcoxon signed rank test (H-l), two-tailed student’s t-test (M,N) or one-way ANOVA test with Sidak multiple comparisons test (P) were applied. Error bars indicate SEM (O) or SD (P). Measure of centrality is mean. * P = < 0.05, ** P = <0.01. Number of replicates are described across conditions from left to right as presented. Heatmap representations of data where asterisks are not present report non-significant changes NES: normalised expression score. Source data

Having established specific changes in redox metabolism driven by truncating mutations in complex I, we next sought to determine the impact of these metabolic alterations in tumor biology. Syngeneic allografts of m.11,944G>A cells, m.12,436G>A cells and wild-type controls were performed subcutaneously in immunocompetent C57/Bl6 mice, establishing tumors in 100% of engraftments. All tumors grew at a rate that reached similar humane endpoints (Fig. 3a), with similar weights and macroscopic histological appearance (Fig. 3b and Extended Data Fig. 3a–c). Bulk measurements of tumor heteroplasmy revealed a subtle, comparable decrease in heteroplasmy of ~10% between engrafted cells and resulting tumors, probably reflecting stroma and immune cell infiltration (Extended Data Fig. 3d), with no consistent change in mtDNA copy number detected at the bulk level (Extended Data Fig. 3e). Measurements of metabolites from m.11,944^60%^ mutant and control tumors revealed an elevated abundance of terminal fumarate adducts succinicGSH and succinylcysteine, characteristic of the metabolic rewiring observed in vitro (Extended Data Fig. 3f). These consistent markers of altered tumor metabolism were coupled to divergent transcriptional signatures between control and mutant tumors (Fig. 3c), with several indicators of altered immune signaling being significantly elevated in mutant tumors compared with controls; notably, allograft rejection, interferon-γ (IFNγ) and interferon-α (IFNα) responses and IL-mediated cytokine signaling gene sets. Higher heteroplasmies correlated with an increased signal in the same gene sets (Extended Data Fig. 3g,h) suggesting a heteroplasmy dose-dependent effect on the immune response. To benchmark these findings against human data, we called somatic mtDNA mutations in the Hartwig Medical Foundation (HMF) metastatic melanoma cohort and stratified patients by mtDNA mutation status into wild-type and >50% variant allele frequency (VAF) groups. This yielded a set of 355 tumor samples (272 wild type, 83 >50% VAF), with 233 having transcriptional profiles. Gene set enrichment analysis (GSEA) revealed consistent transcriptional phenotypes between patient tumors bearing high heteroplasmy pathogenic mtDNA mutations and those identified in our model systems (Fig. 3d and Extended Data Fig. 3i,j). To further dissect these effects, we used whole tumor single-cell RNA sequencing (scRNA-seq) across seven wild-type, three m.12,436^60%^, three m.11,944^60%^ and three m.12,436^80%^ tumors, resulting in 163,343 single-cell transcriptomes. Cells were clustered using Seurat and cellRanger, with preliminary cell ID determined by scType (Fig. 3e,f). Malignant cells were assigned based on low or nil *Ptprc* (CD45) expression, high epithelial score^13^ and aneuploidy determined by copyKAT analysis^14^ (Extended Data Fig. 4a–c). Wild-type tumor single-cell profiles were compared with the grouping of m.11,944^60%^, m.12,436^60%^ and m.12,436^80%^ tumor single-cell profiles for downstream analysis. Consistent with bulk tumor transcriptional profiles, GSEA in malignant cells revealed increased IFNα and IFNγ signatures coupled with decreased glycolysis signatures in high-heteroplasmy tumors (Fig. 3g), which is not observed in vitro before implantation (Extended Data Fig. 3k,l). Downstream regulation of primary metabolic and subsequent immune signaling on malignant cells are also reflected in altered nutrient sensing by mTORC1, transcriptional control of metabolic genes by myc, and tumor necrosis factor alpha signaling (Fig. 3g). Subclustering and GSEA in non-malignant cell clusters revealed similar tumor-wide changes in transcriptional phenotype, with increased IFNα, IFNγ, inflammatory response and IL2-Stat5 signaling again observed (Fig. 3h–k and Extended Data Fig. 4d–k) as well as decreases in oxidative phosphorylation (Fig. 3l and Extended Data Fig. 4l). These indicators of a broad anti-tumor immune response were further supported by decreases in immunosuppressive tumor-resident S100a9^+^ neutrophils, Hmox1^+^ macrophages^15^ and Chil3^+^ monocytes^16^ as well as increases in pro-inflammatory Birc5^+^ (ref. ^17^) and CD74^+^ (ref. ^18^) macrophage populations (Fig. 3m and Extended Data Fig. 4m,n). Taken together, these data demonstrate that in a heteroplasmy-dependent fashion, *Mt-Nd5* mutation is sufficient to remodel the tumor microenvironment and promote an anti-tumor immune response.

### mtDNA mutations sensitize tumors to checkpoint blockade

**Fig. 4:**
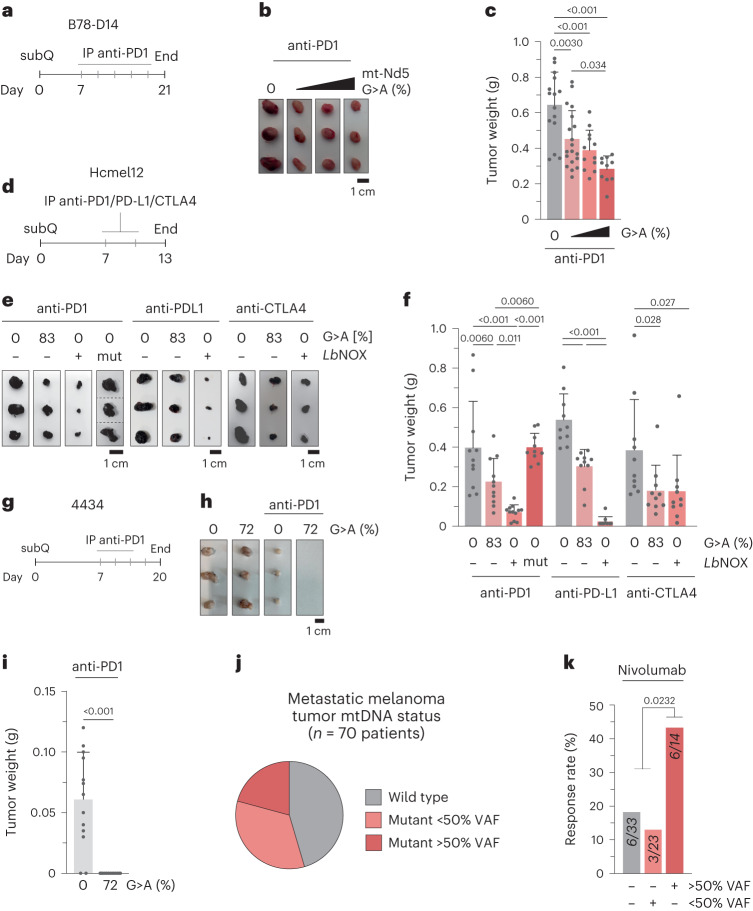
mtDNA mutation and cyto*Lb*NOX-associated microenvironment remodeling sensitizes tumors to checkpoint blockade. **a**, Schematic of the experimental plan and dosing regimen for B78-D14 tumors with anti-PD1 monoclonal antibody (mAb). **b**, Representative images of isolated tumors at day 21. **c**, Tumor weights at day 21 (*n* = 15, 19, 12 and 10 individual tumors). **d**, Schematic of experimental plan and dosing regimen for Hcmel12 tumors with anti-PD1, anti-PD-L1 or anti-CTLA4 mAbs. **e**, Representative images of collected tumors at day 13 for each drug regimen. **f**, Tumor weights at day 13 (*n* = 11, 11, 12 and 10 anti-PD1, *n* = 12 anti-PD-L1 and *n* = 12 anti-CTLA4 individual tumors) for each drug regimen. **g**, Schematic of the experimental plan and dosing regimen for 4434 tumors with anti-PD1 mAb. **h**, Representative images of treated tumors at day 20 and untreated tumors at endpoint. **i**, Tumor weights at day 21 (*n* = 13 individual tumors). **j**, Stratification of a metastatic melanoma patient cohort by mtDNA status. **k**, Response rate of patients to nivolumab by tumor mtDNA mutation status. *P* values were determined using one-way ANOVA with Sidak multiple comparisons test (**c**,**f**), one-tailed Student’s *t*-test (**i**) or chi-squared test (**k**). Error bars, s.d. Measure of centrality is mean. Number of replicates are described across conditions from left to right as presented. Source data

**Extended Data Fig. 5:**
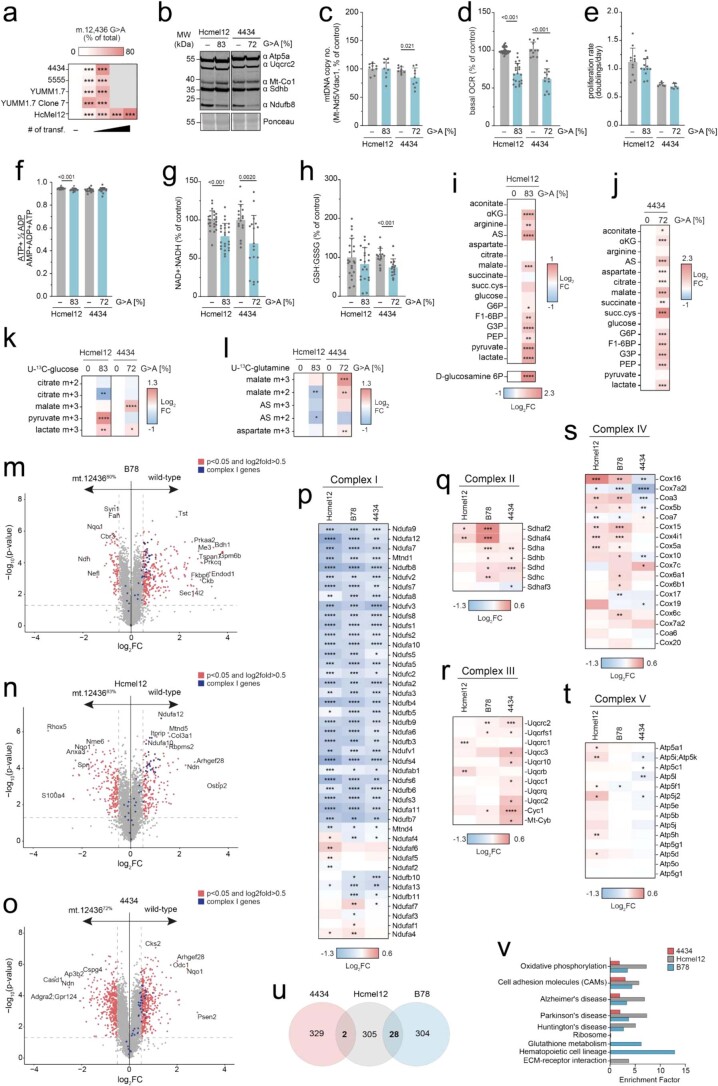
HcMel12 and 4434 mutant cells recapitulate the cellular phenotypes observed in B78-D14 cells. **a** Heteroplasmy changes upon subsequent transfection of melanoma cell lines (n = 3–6 biological replicates). **b** Immunoblot of indicative respiratory chain subunits. Representative result of three biological replicates is shown. **c** mtDNA copy number (n = 9 technical replicates over n = 3 biological replicates). **d** Basal oxygen consumption rate (OCR) (n = 36, 21, 12 and 12 technical replicates over n = 12, 7, 4 and 4 biological replicates) **e** Proliferation rate of cell lines in permissive growth media (n = 12 and 6 technical replicates over n = 4 and 6 biological replicates) **f** Energy (adenylate) charge state (n = 24, 25, 18 and 18 technical replicates over n = 9, 9, 6 and 6 biological replicates). **g** NAD+:NADH ratio (n = 24, 25, 17 and 18 technical replicates over n = 9, 9, 6 and 6 biological replicates). **h** GSH:GSSG ratio (n = 22, 21, 15 and 16 technical replicates over n = 9, 9, 6 and 6 biological replicates). **i** Heatmap of unlabelled steady-state abundance of select metabolites in Hcmel12 cells. Succ. cys, succinylcysteine. (n = 24–25 technical replicates over n = 9 biological replicates). **j** Heatmap of unlabelled steady-state abundance of select metabolites in 4434 cells. Succ. cys, succinylcysteine. (n = 15–18 technical replicates over n = 6 biological replicates). Heatmap of **k** U-^13^C-glucose- and **L** U-^13^C-glutamine-derived select metabolites in Hcmel12 and 4434 cells (n = 6–18 separate technical replicates over n = 2–6 biological replicates). Volcano plot showing detected differences in protein abundance of **m** B78-D14 wild-type versus B78 mt.12436^80%^ cells **N** Hcmel12 wild-type versus Hcmel12 mt.12436^83%^ cells and **o** 4434 wild-type versus 4434 mt.12436^72%^ cells (n = 3 biological replicates). Heatmaps of protein abundances for **p** complex I, **q** complex II, **r** complex III, **S** complex IV and **t** complex V nuclear and mtDNA-encoded subunits in B78 mt.12436^80%^, Hcmel12 mt.12436^83%^ and 4434 mt.12436^72%^ cells compared to their respective wild-type (n = 3 biological replicates). **u** Venn diagram comparing proteomic changes of mutant versus wild-type per cell line. **v** Significant KEGG pathway changes of mutant versus wild-type per cell. A student’s one-tailed t-test (C-H, I-L), student’s two-tailed t-test (P-T), two-tailed Wilcoxon signed rank test (M-O) or Fishers exact test (FDR = 1%) (V) were applied. Error bars indicate SD. Measure of centrality is mean. * P = < 0.05, ** P = <0.01, *** P = <0.001, **** P = <0.0001. Number of replicates are described across conditions from left to right as presented. Heatmap representations of data where asterisks are not present report non-significant changes. Source data

**Extended Data Fig. 6:**
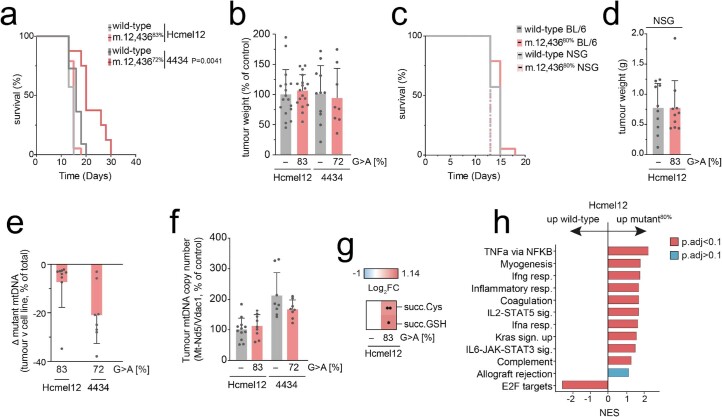
Untreated Hcmel12 and 4334 lineage tumors recapitulate B78-D14 lineage. **a** Survival of C57/BL6 mice subcutaneously injected with indicated cells (n = 8–18 animals). **b** Untreated tumor weight at endpoint (n = 17, 18, 11 and 8 individual tumours). **c** Survival of C57/BL6 and NSG mice subcutaneously injected with indicated Hcmel12 cells (n = 10 animals). **d** Untreated tumor weight at endpoint (n = 10 individual tumours). **e** Change in detected heteroplasmy in bulk tumor samples (n = 14 and 8 individual tumours). 4434 mutant tumors display a modest shift in heteroplasmy that is not seen in Hcmel12 or B78 (Extended Data Fig. 3d), likely reflecting enhanced immunogenicity of the mutant genotype. **f** Bulk tumor mtDNA copy number (n = 12, 9, 8 and 8 individual tumours). **g** Heatmap of steady-state abundance of metabolic terminal fumarate adducts, succinylcysteine and succinicGSH, demonstrating that metabolic changes observed in B78 mutant tumors are preserved in Hcmel12 *in vivo* (n = 9 individual tumours). **h** GSEA of Hcmel12 bulk tumor RNAseq data (n= 3 individual tumours) showing mutant^80%^ versus wild-type. Log-rank (Mantel-Cox) test (A, C), one-tailed student’s t-test applied (B, D) or two-tailed Wilcoxon signed rank test (H) were applied. Error bars indicate SD. Measure of centrality is mean. * P = < 0.05, ** P = <0.01. Number of replicates are described across conditions from left to right as presented. Heatmap representations of data where asterisks are not present report non-significant changes. Source data

Treatment of malignant melanoma can include immune checkpoint blockade (ICB) with monoclonal antibodies against T cell-expressed immune checkpoint receptor PD1, blocking PD-L1 and PD-L2 binding to limit tumor-induced immune tolerance^19^. However, the effectiveness of anti-PD1 treatments and ICB response in patients with melanoma is variable, with a substantial proportion of patients demonstrating limited or no response to treatment while experiencing a poor morbidity profile. Limited efficacy of ICB has previously been linked to immunosuppressive tumor-associated neutrophils^20,21^; therefore, we reasoned that the altered tumor immune microenvironment (TIME) of *Mt-Nd5* mutant tumors could allow for differential sensitivity to ICB, even in an aggressive model of poorly immunogenic melanoma such as B78-D14. This hypothesis was further motivated by the observation that the depleted neutrophil population also demonstrated the highest detected PD-L1 expression in mtDNA mutant tumors (Extended Data Fig. 4o). To this end, we performed further subcutaneous syngeneic allografts of m.12,436^40%^, m.12,436^60%^, m.12,436^80%^, m.11,944^40%^, m.11,944^60%^ and wild-type tumors in immunocompetent animals. Tumors grew untreated for 7 days post graft, and animals were then dosed with a regimen of intraperitoneal anti-PD1 monoclonal antibody every 3 days until the conclusion of the experiment at a fixed point of 21 days (Fig. 4a). A heteroplasmy-defined decrease in tumor weight at endpoint was observed across the mtDNA mutant tumors, with higher mutant heteroplasmies exhibiting greater response to treatment (Fig. 4b,c and Extended Data Fig. 4p), consistent with increased sensitivity of mtDNA mutant tumors to immunotherapy. To further investigate these effects, we produced additional independent models of aggressive murine melanoma, yielding Hcmel12 (Hgf^OE^, Cdk4^R24C^)^22^ cells bearing ~80% m.12,436G>A mutation as well as highly immunogenic 4434 (BRAF^V600E^)^23^ cells bearing ~72% m.12,436G>A mutation (Extended Data Fig. 5a). Both cell lines demonstrated consistent cellular, proteomic and metabolic phenotypes with B78-D14 (Extended Data Fig. 5b–v). Hcmel12 and 4434 mutant and wild-type cells were engrafted into mice, with a similar experimental workflow as previously used (Fig. 4d,g). When untreated, Hcmel12 mutant and wild-type tumors demonstrated comparable time to endpoint and tumor weight at endpoint in both immune-competent and immunocompromised animals (Extended Data Fig. 6a–d). Bulk heteroplasmy, mtDNA copy number, and metabolic and transcriptional profiles of Hcmel12 tumors were similar to those observed in B78-D14 tumors (Extended Data Fig. 6e–h). Untreated 4434 mtDNA mutant tumors demonstrated an extended time to endpoint compared with wild-type 4434 tumors with similar weights at endpoint (Extended Data Fig. 6a,b) probably reflective of the baseline immunogenic state of 4434 tumors when combined with mtDNA mutation, reinforced by the observation that heteroplasmy at endpoint in untreated 4434 mutant tumors was depleted relative to B78-D14 or Hcmel12 (Extended Data Fig. 6e). When anti-PD1 treatment was administered, an enhanced, mtDNA mutation-dependent response was observed in Hcmel12^80%^ mutant tumors (Fig. 4e,f), whereas the immunogenic 4434 tumor model bearing mtDNA mutation underwent complete regression in all subjects (Fig. 4h,i). Enhanced sensitivity to anti-PD-L1 and anti-CTLA4 regimens was also observed in Hcmel^80%^ tumors (Fig. 4e).

### Redox imbalance controls the immunomodulatory effect

**Extended Data Fig. 7:**
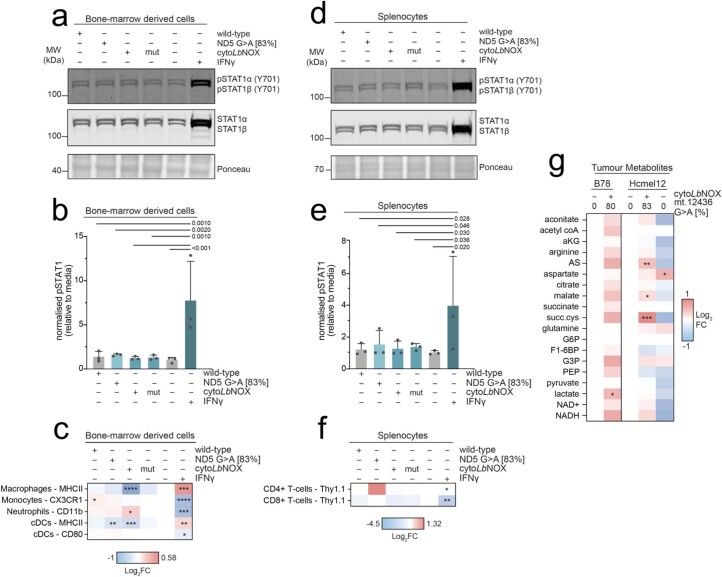
Conditioned media from Hcmel12 cells does not stimulate STAT1 in BMDC and splenocyte cultures. **a** Immunoblot of STAT1 and pSTAT1 isoforms from conditioned BMDCs. Representative image of three biological replicates is shown. **b** Densitometric ratio of normalised pSTAT1 relative to media in BMDC cultures (n = 3 biological replicates). **c** Heatmap of mean fluorescence intensity of specific activation markers of immune cells within BMDC cultures (n = 9 technical replicates over n = 3 biological replicates). Statistics are shown relative to negative control. **d** Immunoblot of STAT1 and pSTAT1 isoforms from conditioned splenocytes. Representative result of three biological replicates is shown. **e** Densitometric ratio of normalised pSTAT1 relative to media in splenocyte cultures (n = 3 biological replicates). **f** Heatmap of mean fluorescence intensity of specific activation markers of immune cells within splenocyte cultures (n = 9 technical replicates over n = 3 biological replicates). Statistics are shown relative to negative control. **g** Heatmap of metabolite abundance changes relative to wild-type tumors for respective tumor lineages (n = 6–38 individual tumours). Macrophages: Cd11b+ Ly6C- F4/80+. Monocytes: CD11b+ Ly6C+ F4/80-. Neutrophils: CD11b+ Ly6C+ Ly6G+. cDCs (conventional Dendritic Cells): CD11c+ MHCII+ F4/80- Ly6C-. All P-values were determined using a one-way ANOVA test with Fisher’s LSD Test (B-C,E-G). Error bars indicate SD. Measure of centrality is mean. * P = < 0.05, ** P = <0.01, *** P = <0.001, **** P = <0.0001. Heatmap representations of data where asterisks are not present report non-significant changes. Source data

**Extended Data Fig. 8:**
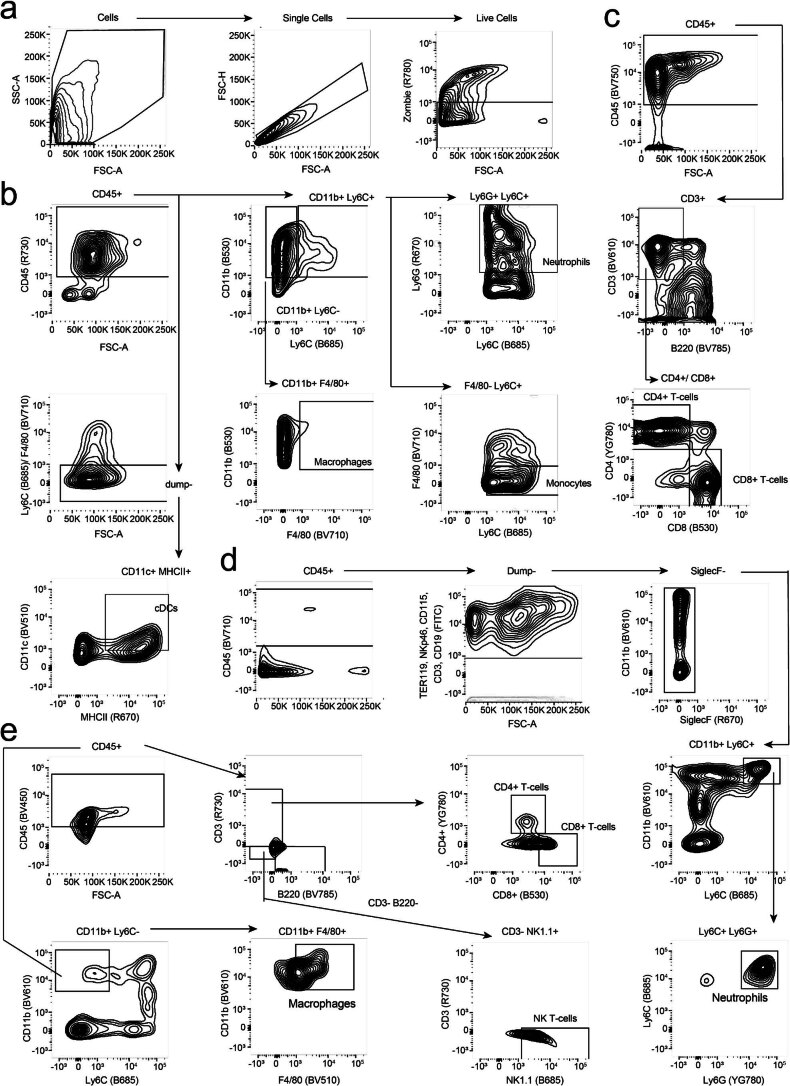
Gating strategy for flow cytometry. **a** Gating strategy for Zombie+ live cells for all experiments. **b** Gating strategy for neutrophils, monocytes, macrophages and cDCs in BMDCs. **c** Gating strategy for CD4+ and CD8+ T-cells in splenocytes. **d** Gating strategy for neutrophils in tumors, lymph nodes and spleens. **e** Gating strategy for CD4+ T-cells, CD8+ T-cells, NK T-cells and macrophages in tumors, lymph nodes and spleens.

**Extended Data Fig. 9:**
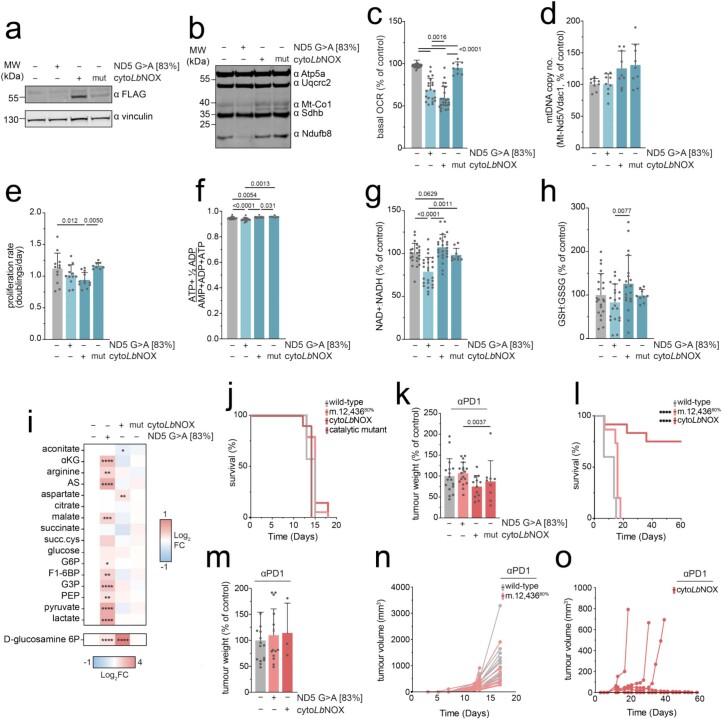
Characterisation and comparison of cytoLbNOX expressing cells in vitro and in vivo. **a** Immunoblot of cyto*Lb*NOX and catalytic mutant expression in clonal population, detected using αFLAG. Representative result of three biological replicates is shown. **b** Immunoblot of indicative respiratory chain subunits. Representative result of three individual runs is shown. **c** Basal oxygen consumption rate (OCR) (n = 36, 21, 24 and 9 technical replicates over n = 12, 7, 8 and 3 biological replicates). A significant decrease is observed in HcMel12 cyto*Lb*NOX, akin to the decrease in basal OCR measured in m.12,436^80%^ cells. **d** mtDNA copy number (n = 9 technical replicates over n = 3 biological replicates). **e** Proliferation rate of cell lines in permissive growth media (n = 12, 12, 12 and 8 technical replicates over n = 4, 4, 4 and 3 biological replicates). **f** Energy (adenylate) charge state (n = 24, 25, 25 and 9 technical replicates over n = 9, 9, 9 and 3 biological replicates). **g** NAD+:NADH ratio (n = 24, 25, 25 and 9 technical replicates over n = 9, 9, 9 and 3 biological replicates). **h** GSH:GSSG ratio (n = 22, 21, 20 and 9 technical replicates over n = 9, 9, 9 and 3 biological replicates). **i** Heatmap of unlabelled steady-state abundance of select metabolites in Hcmel12 cells. Succ. cys, succinylcysteine. (n = 9–25 technical replicates over n = 3–9 biological replicates). **j** Survival of C57/BL6 mice subcutaneously injected with indicated cells (n = 17, 18, 12 and 9 animals). **k** Untreated tumor weight at endpoint (n = 17, 18, 12 and 9 individual tumours). **l** Survival of C57/BL6 mice subcutaneously injected with indicated cells (n = 15, 15 and 10 animals) on sustained anti-PD1 therapy. Only tumors that hit endpoint of 15mm shown for cyto*Lb*NOX. **m** Tumor weight at endpoint for mice on sustained anti-PD1 therapy (n = 15, 15 and 3 individual tumours). Tumor volume changes recorded from injection date for **n** wild-type and m.12436^80%^ (n = 15 individual tumours) and **o** cyto*Lb*NOX tumors (n = 10 individual tumours) on sustained anti-PD1. A one-way ANOVA test with Fisher’s LSD Test (C-I), Log-rank (Mantel-Cox) test (J,L) or one-way ANOVA with Sidak multiple comparisons test (K,M) were applied. Tumor volume calculated as 0.5*L*W^2^ based on calliper measurements. Error bars indicate SD. Measure of centrality is mean. * P = < 0.05, ** P = <0.01, *** P = <0.001, **** P = <0.0001. Number of replicates are described across conditions from left to right as presented. Heatmap representations of data where asterisks are not present report non-significant changes. Source data

**Extended Data Fig. 10:**
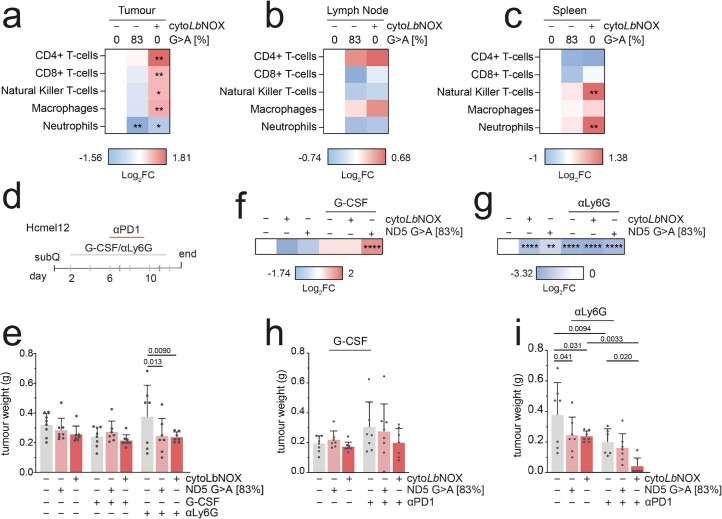
Sensitisation to anti-PD1 negatively correlates to abundance of tumour-resident neutrophils. Abundance of specific immune cells in **a** tumor, **b** tumour-draining lymph node and **c** spleen in untreated mice (n = 4–8 individual samples). **d** Schematic of the experimental plan and dosing regimen for Hcmel12 tumors with anti-PD1 monoclonal antibody (mAb) and either G-CSF or anti-Ly6G. **e** Tumor weight of untreated mice compared to mice treated with G-CSF or anti-Ly6G (n = 8, 8, 7, 8, 7, 8, 7, 7, 7 and 7 individual tumours). Log_2_ fold change of tumor neutrophils in untreated and treated mice relative to untreated control for **f** G-CSF and **g** anti-Ly6G (n = 4–8 individual tumours). Tumor weight of mice treated with anti-PD1 or anti-PD1 and **h** G-CSF (n = 8, 7, 8, 8, 8, 7 and 7 individual tumours) or **i** anti-Ly6G (n = 8, 8, 8, 7, 8 and 8 individual tumours). Natural Killer T-cells: CD4- CD8- NK1.1+. Macrophages: Cd11b+ Ly6C- F4/80+. Neutrophils: CD11b+ Ly6C+ Ly6G+. All P-values were determined using a one-way ANOVA test with Fisher’s LSD Test (A-C,E-I). Error bars indicate SD. Measure of centrality is mean. * P = < 0.05, ** P = <0.01, **** P = <0.0001. Number of replicates are described across conditions from left to right as presented. Heatmap representations of data where asterisks are not present report non-significant changes. Source data

It has been shown previously that immune cell proliferation and activation can be regulated by metabolite composition^24^. To determine whether the enhanced ICB response owing to the altered TIME was driven by cell non-autonomous metabolic or secreted factors at the primary site, we used conditioned medium transfer with bone marrow-derived cells and splenocyte cultures, demonstrating no substantive differential activation of immune signaling for mtDNA mutant-derived conditioned medium (Extended Data Figs. 7 and 8a–c). To further investigate the underlying sensitization, we next modified wild-type Hcmel12 cells to reproduce key elements of the high heteroplasmy mutant *Mt-Nd5*-associated phenotype—specifically, an altered redox state and decreased basal OCR—by constitutively expressing cyto*Lb*NOX or a catalytic mutant of the enzyme (Extended Data Fig. 9a–i). When grafted into mice, Hcmel12 cyto*Lb*NOX and catalytic mutant tumors demonstrated comparable time to endpoint and tumor weight at endpoint as wild-type or *Mt-Nd5* mutant tumors (Extended Data Fig. 9j,k). When challenged with anti-PD1 treatment, Hcmel12 cyto*Lb*NOX tumors recapitulate the response of Hcmel12 *Mt-Nd5* m.12,436^80%^ tumors, whereas catalytic mutant tumors were unresponsive (Fig. 4e,f). Taken together with metabolic measurements demonstrating subtle changes in the metabolite profiles of mtDNA mutant tumors and a lack of consonance between these and the cyto*Lb*NOX tumor metabolic profile (Extended Data Fig. 9i), these data suggest that primary site metabolite composition is not a direct mediator of the anti-tumor immune response under these conditions. Furthermore, these data support the notion that altered cellular redox poise, in the absence of broader metabolic changes associated with mtDNA mutation, is sufficient to sensitize tumors to ICB. Treatment of Hcmel12 wild type, m.12436^80%^ and cyto*Lb*NOX tumors with anti-PD1 to an extended humane endpoint demonstrated limited survival extension of m.12436^80%^ tumor-bearing mice, whereas the majority of cyto*Lb*NOX tumors underwent complete regression (Extended Data Fig. 9l–o). Sensitization to anti-PD1 was dependent on and negatively correlated with the proportion of tumor-resident neutrophils, as manipulated by tumor neutrophil-increasing granulocyte colony-stimulating factor treatment or neutrophil-depleting anti-Ly6G treatment (Extended Data Figs. 8a,d–e and 10). To benchmark these findings from mice against clinical data, we re-analyzed a previously reported, well-characterized cohort of patients with treatment-naive metastatic melanoma who received the anti-PD1 monoclonal antibody nivolumab^25^. By identifying mtDNA mutant cancers and stratifying this patient cohort solely based on cancer mtDNA mutation status (Fig. 4j), the 70 patients in this cohort were divided into three groups: mtDNA wild type (33), <50% VAF (23) and >50% VAF (14). The cancer mtDNA mutation-status-naive cohort response rate was 22% for partial or complete responses to nivolumab; however, the rate of response for >50% mtDNA mutation VAF cancers was 2.6-fold greater than wild-type or <50% VAF cancers (Fig. 4k).

## Discussion

These data confirm that somatic mtDNA mutations, commonly observed in human tumors, can exert direct effects on cancer cell metabolic phenotypes. In contrast with clinically presented mitochondrial disease owing to germline mtDNA mutation^2^, tumor mtDNA mutations are able to exert effects at a comparably low heteroplasmic burden and without dramatically impacting energy homeostasis. The direct link we observed between redox perturbations and enhanced glycolytic flux subtly alters our view of mtDNA mutation to a potentially adaptive gain-of-function rather than an exclusively loss-of-function event, and the discovery that cancer-relevant mtDNA mutations can underpin aerobic glycolysis in cancer cells warrants further assessment of the relationship between classical Warburg metabolism^26^ and mtDNA mutation status.

In addition to cell-autonomous metabolic impacts, the data here reveal that a functional consequence of somatic mtDNA mutation in tumor biology is a remodeling of the TIME, mediating therapeutic susceptibility to ICB. Although we have demonstrated that tumor-resident neutrophils are a key mediator of the immunomodulatory impact of redox perturbation, it seems likely that the loss of neutrophils in redox-modified tumors is a secondary effect owing to T cell activation and proliferation, influencing neutrophil recruitment to the tumor and subsequent activity in the draining lymph node^27^. It has been shown previously that the mutational burden of mtDNA is not correlated with nuclear DNA tumor mutational burden^1^. Thus, enhanced sensitivity to ICB owing to mtDNA mutation, in the context of both immunogenic and non-immunogenic models of melanoma, supports the notion that cancer cell-specific redox perturbation acts as an independent, sensitizing effector of ICB, with synergistic potential. Truncating mutations in mtDNA, analogous to those described here, affect ~10% of all cancers regardless of tissue lineage, with non-truncating, pathogenic mtDNA mutations presenting in a further 40–50% of all cancers^3^. A broad influence over the anti-tumor immune response in these cancers might also be expected.

In addition to the exploitation of mtDNA mutant tumor vulnerability and stratification of patients for ICB, the data presented here suggest that the ICB response-governing effects observed are principally cancer-cell-intrinsic and redox-state-mediated in nature. Recreating such states in mtDNA wild-type cancer, for both immune-responsive and non-responsive tumor types, could therefore also be of therapeutic benefit.

## Methods

Our research complies with all relevant ethical regulations. Animal experiments were carried out in accordance with the UK Animals (Scientific Procedures) Act 1986 (P72BA642F) and by adhering to the ARRIVE guidelines with approval from the local Animal Welfare and Ethical Review Board of the University of Glasgow. Further information on research design is available in the Nature Research Reporting Summary linked to this article.

### Maintenance, transfection and FACS of cell lines

B78 melanoma cells (RRID: CVCL_8341), Hcmel12 cells^22^ and 4434 cells^24^ were maintained in DMEM containing GLUTAMAX, 0.11 g l^−1^ sodium pyruvate and 4.5 g l^−1^ d-glucose (Life Technologies), supplemented with 1% penicillin–streptomycin (Life Technologies) and 10% FBS (Life Technologies). Cells were grown in incubators at 37 °C and 5% CO_2_. Cells were transfected using Lipofectamine 3000 (Life Technologies) using a ratio of 5 µg DNA:7.5 µl Lipofectamine 3000. Cells were sorted as previously described^28^ and thereafter grown in the same base DMEM media supplemented with 20% FBS and 100 µg m^−1^ of uridine (Sigma).

B78 melanoma cells were sourced from ATCC. Hcmel12, YUMM1.7 Clone 7, YUMM1.7, 4434 and 5555 melanoma cells were gifted by A. Viros (Cancer Research UK Manchester Institute).

### Use of animal models

Mice were housed in conventional cages in an animal room at a controlled temperature (19–23 °C) and humidity (55 ± 10%) under a 12 h light:12 h dark cycle. Experiments used male C57BL/6 or NOD scid gamma mice at ~8 weeks of age that were injected subcutaneously with either 2.5 × 10^5^ B78 cells or 1 × 10^4^ HcMel12 cells, both prepared in 1:1 RPMI (Life Technologies) and Matrigel (Merck). For 4434 cells, 3 × 10^6^ cells were prepared in PBS and injected subcutaneously. Untreated mice were killed at an endpoint of 15 mm tumor measurement, which was not exceeded for any experiment. Mice receiving immunotherapy were killed at a fixed timepoint of day 21, 13 or 20 for tumors derived from B78-D14, Hcmel12 or 4434 cells, respectively.

For immunotherapy experiments, mice were put on a dosing regimen of 200 µg of anti-PD1 given intraperitoneally twice per week. The first dose was given 7 days post injection and all mice were killed at 21 or 13 days post injection for B78 or HcMel12 cells, respectively.

Either 5 µg mouse recombinant granulocyte colony-stimulating factor (Stemcell) or 100 µg anti-mouse Ly6G–clone 1A8 (2B Scientific) was given intraperitoneally every 2 days post engraftment to mice for neutrophil depletion experiments.

### Construction of DdCBE plasmids

TALEs targeting mt.12,436 and mt.11,944 were constructed with advice from B. Mok and D. Liu (Broad Institute, USA). TALEs were synthesized (Thermo Fisher GeneArt) and assembled as illustrated in Fig. 1a, with the left TALEs being cloned into pcDNA3.1^−^_mCherry^29^ and the right into pTracer CMV/Bsd^29^, allowing for the co-expression of mCherry and GFP, respectively.

### Pyrosequencing assay

DNA was extracted from cell pellets using the DNeasy Blood & Tissue Kit (Qiagen) as per the manufacturer’s instructions. PCR was then performed using the PyroMark PCR Mix (Qiagen) for 50 cycles with an annealing temperature of 50 °C and an extension time of 30 s. PCR products were run on the PyroMark Q48 Autoprep (Qiagen) as per the manufacturer’s instructions.

### Statistical analysis of human melanoma genomic data

Figures and statistical analysis for human subject data for HMF and IMPACT cohorts were conducted in the R statistical programming environment (v.1.4.1717). Figures were generated using the ggplot2 library. For proportions, 95% confidence intervals were calculated using the Pearson–Klopper method, and rates were calculated by Poisson’s exact test. *P* values represent comparisons from Fisher’s exact test.

### mtDNA sequencing

Cellular DNA was amplified to create two ~8 kbp overlapping mtDNA products using PrimeStar GXL DNA Polymerase (Takara Bio) as per the manufacturer’s instructions. Resulting amplicons were sequenced using the Illumina Nextera kit (150 cycle, paired-end). To determine the percentage of non-target C mutations in mtDNA, we first identified all C and G nucleotides with adequate sequencing coverage (>1000×) in both the reference and experimental samples. Then, for each of the four experimental samples, we identified positions for which sequencing reads in the experimental sample corresponded to G>A or C>T mutations. We further filtered the resulting list of mutations to retain only those with a heteroplasmy over 2% and removed mutations that were also present in control samples. Finally, the non-target percentage was calculated as the fraction of total possible C or G positions that were mutated.

### Proteomics methodology

#### Sample preparation

Cells were lysed in a buffer containing 4% SDS in 100 mM Tris-HCl pH 7.5 and 55 mM iodoacetamide. Samples were then prepared as previously described^30^, with minor modifications. Alkylated proteins were digested first with endoproteinase Lys-C (1:33 enzyme:lysate) for 1 h, followed by overnight digestion with trypsin (1:33 enzyme:lysate). Digested peptides from each experimental condition and a pool sample were differentially labeled using TMT16-plex reagent (Life Technologies) as per the manufacturer’s instructions. Fully labeled samples were mixed in equal amounts and desalted using 100 mg Sep Pak C18 reverse-phase solid-phase extraction cartridges (Waters). TMT-labeled peptides were fractionated using high-pH reverse-phase chromatography on a C18 column (150 × 2.1 mm internal diameter; Kinetex EVO (5 μm, 100 Å)) on a high-performance liquid chromatography system (LC 1260 Infinity II, Agilent). A two-step gradient was applied, 1% to 28% B (80% acetonitrile) over 42 min, then from 28% to 46% B over 13 min to obtain a total of 21 fractions for mass spectrometry analysis.

#### Ultra-high-performance liquid chromatography–tandem mass spectrometry

Peptides were separated by nanoscale C18 reverse-phase liquid chromatography using an EASY-nLC II 1200 (Life Technologies) coupled to an Orbitrap Fusion Lumos mass spectrometer (Life Technologies). Elution was carried out using a binary gradient with buffer A (water) and buffer B (80% acetonitrile), both containing 0.1% formic acid. Samples were loaded with 6 µl of buffer A into a 50 cm fused silica emitter (New Objective) packed in-house with ReproSil-Pur C18-AQ, 1.9 μm resin (Dr Maisch GmbH). The packed emitter was kept at 50 °C by means of a column oven (Sonation) integrated into the nanoelectrospray ion source (Life Technologies). Peptides were eluted at a flow rate of 300 nl min^−1^ using different gradients optimized for three sets of fractions: 1–7, 8–15 and 16–21 (ref. ^30^). Each fraction was acquired for a duration of 185 min. Eluting peptides were electrosprayed into the mass spectrometer using a nanoelectrospray ion source (Life Technologies). An active background ion reduction device (ESI Source Solutions) was used to decrease the air contaminants signal level. Xcalibur software (Life Technologies) was used for data acquisition. A full scan over mass range of 350–1400 *m*/*z* was acquired at 60,000 resolution at 200 *m*/*z*, with a target value of 500,000 ions for a maximum injection time of 50 ms. Higher energy collisional dissociation fragmentation was performed on the most intense ions during 3 s cycle time, for a maximum injection time of 120 ms or a target value of 100,000 ions. Peptide fragments were analyzed in the Orbitrap at 50,000 resolution.

#### Data analysis

The mass spectrometry raw data were processed with MaxQuant software^29^ (v.1.6.1.4) and searched with the Andromeda search engine^31^, querying SwissProt^32^ *Mus musculus* (25,198 entries). First and main searches were performed with precursor mass tolerances of 20 ppm and 4.5 ppm, respectively, and a tandem mass spectrometry (MS/MS) tolerance of 20 ppm. The minimum peptide length was set to six amino acids and specificity for trypsin cleavage was required, allowing up to two missed cleavage sites. MaxQuant was set to quantify on ‘Reporter ion MS2’, and TMT16plex was set as the isobaric label. Interference between TMT channels was corrected by MaxQuant using the correction factors provided by the manufacturer. The ‘filter by PIF’ option was activated and a ‘reporter ion tolerance’ of 0.003 Da was used. Modification by iodoacetamide on cysteine residues (carbamidomethylation) was specified as variable, as well as methionine oxidation and amino-terminal acetylation modifications. The peptide, protein and site false discovery rate (FDR) was set to 1%. The MaxQuant output ProteinGroup.txt file was used for protein quantification analysis with Perseus software^33^ (v.1.6.13.0). The datasets were filtered to remove potential contaminants and reverse peptides that match the decoy database and proteins only identified by site. Only proteins with at least one unique peptide that were quantified in all replicates in at least one experimental group were used for analysis. Missing values were added separately for each column. The TMT-corrected intensities of proteins were normalized first by the median of all intensities measured in each replicate and then by using the LIMMA plugin^34^ in Perseus. Significantly regulated proteins between two groups were selected using a permutation-based Student’s *t*-test, with an FDR set at 1%.

### Protein extraction and measurement

Cell pellets were lysed in RIPA buffer (Life Technologies) supplemented with cOmplete Mini Tablets and cOmplete Mini Protease Inhibitor Tablets (Roche). Samples were incubated on ice for 20 min and then spun at 14,000×*g* for 20 min. The isolated supernatant containing total cellular protein was then quantified using a DC Protein Assay (Bio-Rad Laboratories) performed as per the manufacturer’s instructions.

### Immunoblotting

To detect protein by western blotting, 60 µg of protein was resolved on SDS–PAGE 4–12% Bis-Tris Bolt gels (Life Technologies). Protein was transferred onto a nitrocellulose membrane using a Mini Trans-Bolt Cell (Bio-Rad Laboratories). Membranes were then stained with Ponceau S Staining Solution (Life Technologies) to measure loading before overnight incubation with the primary antibody prepared in 5% milk in 1× TBST. Imaging was performed using the Odyssey DLx imaging system (Licor).

Antibodies used were Total OXPHOS Rodent WB antibody cocktail (1:800; ab110413, Abcam), Monoclonal Anti-FLAG M2 antibody (1:1000; F1804, Sigma) and Recombinant anti-vinculin antibody (1:10,000; ab129002, Abcam).

### Mitochondrial isolation

Cells were grown in Falcon Cell Culture 5-layer Flasks (Scientific Laboratory Supplies) and grown to near 100% confluency. Cells were then collected, and mitochondria were extracted as previously described^35^.

### Blue-native PAGE

Isolated mitochondria were solubilized in 1× NativePage Sample Buffer supplemented with 1% digitonin (Life Technologies). Samples were incubated on ice for 10 min and then centrifuged at 20,000×*g* for 30 min at 4 °C. Supernatants were isolated and total extracted protein was quantified using the DC Protein Assay (Bio-Rad Laboratories). Samples were prepared and run on NativePage 4–12% Bis-Tris gels as per the manufacturer’s instructions (Life Technologies). For immunoblotting, samples were transferred onto PVDF membranes using a Mini Trans-Bolt Cell (Bio-Rad Laboratories). Subsequent probing and imaging was performed as described above for immunoblotting. Loading was visualized using Coomassie Blue on a duplicate gel.

In-gel assays were performed for complex I and II activity as previously described^35^.

### Digital droplet PCR

Samples were prepared in triplicate in a 96-well plate using 1 ng of DNA, 100 nM of each primer, 10 µl of QX200 ddPCR EvaGreen Supermix and water to 20 µl. Droplet generation, PCR and measurements were then performed on the QX200 Droplet Digital PCR System (Bio-Rad Laboratories) as per the manufacturer’s instructions, with the primer annealing temperature set at 60 °C.

### Seahorse assay

The Seahorse XF Cell Mito Stress Test (Agilent) was performed as per the manufacturer’s instructions. In brief, cells were plated into a Seahorse 96-well plate at 2 × 10^4^ cells per well 1 day before the assay. A sensor cartridge was also allowed to hydrate in water at 37 °C overnight. The water was replaced with Seahorse XF Calibrant and the sensor cartridge was re-incubated for 45 min. Oligomycin, FCCP, rotenone and antimycin A were then added to their respective seahorse ports to a final concentration of 1 µM in the well before sensor calibration on the Seahorse XFe96 Analyzer (Agilent). Meanwhile, cell media was replaced with 150 µl Seahorse XF Media supplemented with 1% FBS, 25 mM glucose, 1 mM sodium pyruvate and 2 mM glutamine and incubated at 37 °C for 30 min. The cell plate was then inserted into the analyzer post calibration and run.

For read normalization, protein extraction and measurement was performed as described above.

### Mitochondrial membrane potential and pH gradient

Membrane potential and pH gradient were measured using multi-wavelength spectroscopy as previously described^36,37^. In brief, cultured cells were disassociated by gentle tapping and then spun down and re-suspended at a density of 1 × 10^7^ cells per ml in FluroBrite supplemented with 2 mM glutamine in a temperature-controlled chamber. Changes in mitochondrial cytochrome oxidation states were then measured with multi-wavelength spectroscopy. The baseline oxidation state was measured by back-calculation using anoxia to fully reduce the cytochromes, and a combination of 4 μM FCCP and 1 μM rotenone was used to fully oxidize the cytochromes. The membrane potential was then calculated from the redox poise of the b-hemes of the *bc*_1_ complex, and the pH gradient was measured from the turnover rate and redox span of the *bc*_1_ complex using a model of turnover^37^.

### Mitochondrial NADH oxidation state

Changes in NAD(P)H fluorescence were measured simultaneously with mitochondrial membrane potential using 365 nm excitation. The resultant emission spectrum was then measured with multi-wavelength spectroscopy^36^. The baseline oxidation state of the mitochondrial NADH pool was back-calculated using anoxia to fully reduce and 4 μM FCCP to fully oxidize the mitochondrial NADH pool, respectively, assuming the cytosolic NADH pool and NADPH pools did not change with these interventions and short time period.

### In vitro metabolomics

Cells were seeded 2 days before metabolite extraction to achieve 70–80% confluency on the day of extraction. The following day, cells were replenished with excess fresh media to prevent starvation at the point of extraction. For steady-state experiments, media was prepared as described above with the substitution of GLUTAMAX with 2 mM l-glutamine. For U-^13^C-glucose and 4-^2^H_1_-glucose isotope tracing experiments, media was prepared as follows: DMEM, no glucose (Life Technologies) supplemented with 0.11 g l^−1^ sodium pyruvate, 2 mM l-glutamine, 20% FBS, 100 µg m^−1^ uridine and 25 mM glucose isotope (Cambridge Isotopes). For isotope tracing experiments using U-^13^C-glutamine and 1-^13^C-glutamine, DMEM containing 4.5 g l^−1^ d-glucose and 0.11 g l^−1^ sodium pyruvate was supplemented with 20% FBS, 100 µg ml^−1^ uridine and 4 mM glutamine isotope (Cambridge Isotopes).

On the day of extraction, 20 µl of media was added to 980 µl of extraction buffer from each well. Cells were then washed twice with ice-cold PBS. Extraction buffer (50:30:20, v/v/v, methanol/acetonitrile/water) was then added to each well (600 µl per 2 × 10^6^) and incubated for 5 min at 4 °C. Samples were centrifuged at 16,000×*g* for 10 min at 4 °C and the supernatant was transferred to liquid chromatography–mass spectrometry (LCMS) glass vials and stored at −80 °C until run on the mass spectrometer.

Mass spectrometry and subsequent targeted metabolomics analysis was performed as previously described^38^ using Tracefinder (v.5.1). Compound peak areas were normalized using the total measured protein per well quantified with a modified Lowry assay^38^.

### In vitro measurements of fumarate

Samples were prepared as described above.

Fumarate analysis was carried out using a Q Exactive Orbitrap mass spectrometer (Thermo Scientific) coupled to an Ultimate 3000 high-performance liquid chromatography system (Thermo Fisher Scientific). Metabolite separation was done using a HILIC-Z column (InfinityLab Poroshell 120, 150 × 2.1 mm, 2.7 µm, Agilent) with a mobile phase consisting of a mixture of A (40 mM ammonium formate, pH 3) and B (90% ACN / 10% 40 mM ammonium formate). The flow rate was set to 200 µl min^−1^ and the injection volume was 5 µl. The gradient started at 10% A for 2 min, followed by a linear increase to 90% A for 15 min; 90% A was then maintained for 2 min, followed by a linear decrease to 10% A for 2 min and a final re-equilibration step with 10% A for 5 min. The total run time was 25 min. The Q Exactive mass spectrometer was operated in negative mode with a resolution of 70,000 at 200 *m/z* across a range of 100 to 150 *m/z* (automatic gain control) target of 1 × 10^6^ and maximum injection time of 250 ms. Subsequent targeted metabolomics analysis was performed as previously described^38^ using Tracefinder (v.5.1). Compound peak areas were normalized using the total measured protein per well quantified with a modified Lowry assay^38^.

### siRNA knockdown for metabolomics

A total of 1.2 × 10^4^ cells were plated into 12-well cell culture plates and incubated at 37 °C and 5% CO_2_ overnight. The following day, cells were transfected with 5 µl of 5 µM siRNA with 5 µl of DharmaFECT 1 Transfection Reagent (Horizon Discovery). Cells were either transfected with ON-TARGETplus MDH1 siRNA (L-051206-01-0005; Horizon Discovery) or ON-TARGETplus non-targeting control siRNA (D-001810-10-05; Horizon Discovery). Cells were supplemented with excess media the following day and metabolites were extracted 48 h post transfection as outlined above. Mass spectrometry and subsequent targeted metabolomics analysis was performed as previously described^38^, using Tracefinder (v.5.1). Compound peak areas were normalized using the total measured protein per well quantified with a modified Lowry assay^38^.

### LbNOX treatment for metabolomics

pUC57-LbNOX (Addgene, no. 75285) and pUC57-mitoLbNOX (Addgene, no. 74448) were gifts from V. Mootha. Both enzyme sequences were amplified using Phusion PCR (Life Technologies) as per the manufacturer’s instructions. These products were cloned into pcDNA3.1^−^_mCherry^29^ through the *NheI* and *BamHI* restriction sites and used for subsequent experiments.

Cells were transfected and sorted as described above, and 3 × 10^4^ mCherry^+^ cells were plated per well into a 12-well plate. Cells were allowed to recover overnight at 37 °C and 5% CO_2_ followed by the addition of excess media to each well. Metabolites were extracted the following day and analyzed as outlined above. Mass spectrometry and subsequent targeted metabolomics analysis were performed as previously described^38^ using Tracefinder (v.5.1). Compound peak areas were normalized using the total measured protein per well quantified with a modified Lowry assay^38^.

### Bulk tumor metabolomics

Tumor fragments (20–40 mg) were flash-frozen on dry ice when collected. Metabolites were extracted using the Precellys Evolution homogenizer (Bertin) with 25 µl of extraction buffer per mg of tissue. Samples were then centrifuged at 16,000×*g* for 10 min at 4 °C and the supernatant was transferred to LCMS glass vials and stored at −80 °C until analysis.

Samples were run and subsequent targeted metabolomics analysis was performed as previously described^38^ using Tracefinder (v.5.1). Compound peak areas were normalized using the mass of the tissue.

### IC_50_ measurements

Cells were plated in a 96-well plate at 500 cells per well in 200 µl of cell culture media. The following day, the media was replaced with 0–100 mM 2-deoxyglucose, 0–1 M metformin, 0–10 µM rotenone and 0–1 mM oligomycin in quadruplicate. Plates were imaged once every 4 h on an IncuCyte Zoom (Essen Bioscience) for 5 days. Final confluency measurements were calculated using the system algorithm and the IC_50_ was determined by GraphPad Prism.

### Hematoxylin and eosin staining

Hematoxylin and eosin staining and slide scanning were performed as previously described^39^.

### Bulk cell and tumor RNA-seq

#### RNA extraction and sequencing

Cell pellets were stored at −80 °C. Tumor fragments (20–40 mg) were stored in RNAlater (Sigma) at −80 °C. Samples were sent to GeneWiz Technologies for RNA extraction and sequencing.

#### Data processing

Bulk RNA-seq reads were aligned against the mouse reference genome GRCm39 using STAR two-pass alignment (v.2.7.10a) with default settings^40^. FeatureCounts from the subread package (v.2.0.3) using genomics annotations in GTF format (Mus_musculus.GRCm39.107.gtf) were used to derive the gene count matrix^41^. Counts were normalized to gene length and library size using the weighted trimmed mean of M-values method in the edgeR package (v.3.40.1)^42^.

#### Differential gene expression

Differential gene expression tests were applied by using limma-voom in the limma package (v.3.54.0)^34,43^. Significant differential expression was set to an FDR-adjusted *P* value of <0.1.

#### Gene set enrichment analysis

Genes are pre-ranked by the sign of the log_2_ fold changes between experimental and control conditions multiplied by −log_10_ of the *P* value derived from the differential gene expression test calculated by limma-voom. Pre-ranked gene lists and Hallmark pathway gene sets (mh.all.v2022.1.Mm.symbols.gmt) were used as the input for the fgsea function in the fgsea R package (v.1.22.0) to perform the GSEA^44^.

### scRNA-seq methodology

#### Tumor preparation

Tumors were dissected into small pieces and re-suspended in digestion buffer (RPMI (Gibco) containing 100 U ml^−1^ Collagenase IV (Sigma), 500 U ml^−1^ Collagenase II (Sigma) and 0.2 mg ml^−1^ DNase I (Sigma)). Samples were incubated on a shaker at 37 °C for 40 min and vigorously shaken at the 20 and 40 min mark. Samples were passed through a 70 µM cell strainer (Fisher Scientific) and pelleted at 800×*g* for 2 min. Samples were re-suspended in FACS buffer (PBS containing 2% FBS (Gibco)) with 1 µg ml^−1^ DAPI stain (Life Technologies). Approximately 100,000 live cells were sorted into PBS + 0.04% BSA.

#### Processing of samples for sequencing

Single-cell suspensions were processed through a 10× Genomics Chromium controller using the Single Cell Gene Expression kit (10× Genomics, Chromium Next GEM Single Cell 3′ Kit v.3.1) to generate emulsions, which were first reverse-transcribed and then PCR-amplified to generate cDNA. Sequencing libraries were then generated using 10 µl of cDNA as outlined in the 10× Genomics protocol (CG000315 Rev C). In brief, cDNA was first fragmented, end-repaired and adaptors ligated, followed by PCR amplification and size selection to generate final libraries, which were sequenced on a NovaSeq S4 flowcell (Illumina) to a depth of 25,000 reads per cell.

#### Pre-processing of scRNA transcriptomics data, batch effect correction and clustering

CellRanger (v.7.0.1) was used to map the reads in the FASTQ files to the mouse reference genome (GRCm39)^45^. The Seurat (v.4.2.0) package in R (v.4.2.1) was used to handle the pre-processed gene count matrix generated by cellRanger^46^. As an initial quality-control step, cells with fewer than 200 genes as well as genes expressed in less than three cells were filtered out. Cells with >5% mitochondrial counts, unique molecular identifier counts of >37,000 and gene counts of <500 were then filtered out, resulting in 163,343 cells. The filtered gene counts matrix (31,647 genes and 127,356 cells) was normalized with the NormalizeData function using the log(Normalization) method and scale.factor to 10,000. The FindVariableFeatures function was used to identify 2,000 highly variable genes for principal component analysis. The first 50 principal components were selected for downstream analysis. The RunHarmony function from the harmony package (v.0.1.0) with default parameters was used to correct batch effects^47^. The RunUMAP function with the reduction from ‘harmony’ was used to generate uniform manifold approximation and projection (UMAP) plots for cluster analysis. The FindClusters function was used with the resolution parameter set to 0.2.

The clustifyr package in R (v.1.8.0) was used to calculate the Pearson correlation coefficient between the average gene expression of each cluster and the reference data containing 253 sorted mouse immune cells (ref_immgen) from clustifyrdatahub (v.1.6.0)^48^. The Pearson correlation coefficient threshold was set to 0.53 by clustifyr. We manually reviewed the differentially expressed genes in each cluster using the FindAllMarkers functions with parameters only.pos=TRUE, min.pct=0.25 and logfc.threshold=0.25 set to well-known marker genes to further adjust the cell type assignments. This included Ptprc for pan-immune cells, Cd3e for pan-T cells, Ncr1 for natural killer cells, Siglech for plasmacytoid dendritic cells, CD79a for B cells, Kit for mast cells, Csf3r for neutrophils, Xcr1, Itgax for conventional dendritic cells and CD14 for pan-myeloid cells. Clusters that did not contain more than 50% tumor cells or cells expressing Ptprc were filtered out, yielding a final 15 clusters.

T cell and natural killer cell sub-populations were classified by first extracting these 9,746 cells from the analysis and then following the same steps in Seurat as above with the resolution set to 0.6 for the FindClusters function. In this case, the Pearson correlation coefficient threshold was set to 0.54 by clutifyr. Clusters identified as other immune cells were filtered out, yielding a final 11 clusters. Sub-populations of macrophages, monocytes, dendritic cells, plasmacytoid dendritic cells, B cells, neutrophils and mast cells (84,241 cells) were classified in a similar manner, with the Pearson correlation coefficient threshold set to 0.58 by clustifyr in this case. This analysis yielded a further 19 clusters.

#### Epithelial score

Average gene expression from cytokeratins, Epcan, and Sfn were used to calculate the epithelial score.

#### Single-cell copy number estimation

CopyKAT (v.1.1.0) was used to estimate the copy number status of each cell^14^. Parameters were set as ngene.chr=5, win.size=25, KS.cut=0.1, genome = ’mm10’ and cells annotated as T cells or natural killer cells in the UMAP as diploid reference cells.

#### Identification of differentially expressed marker genes

Top differentially expressed genes in each cluster were identified using the FindAllMarkers function in the Seurat R package. Parameters for expression difference were set to be at least 1.25 times fold changes (logfc.threshold=1.25) and an adjusted *P* value of <0.05, with gene expression detected in at least 10% of cells in each cluster (min.pct=0.1). The top 20 highly differentially expressed genes in each cluster ranked by average fold change were defined as marker genes.

#### Pathway enrichment analysis of single-cell transcriptomics data

For cells in each identified cluster in the UMAP, the wilcoxauc function from the presto R package (v.1.0.0) was used to conduct a Wilcox rank-sum test to obtain the fold change and *P* value for all genes between cells in the high heteroplasmy group for both mutations and control group^49^. The genes were ranked in decreasing order according to the formula sign(log_2_(fold change)) × (−log_10_(*P* value)). This ranked gene list and mouse hallmark pathways (mh.all.v2002.1.Mm.symbols.gmt) from the MSigDB database were used as inputs for GSEA using the fgsea function from fgsea R package (v.1.22.0) with parameters of eps=0, minSize=5 and maxSize=500 (ref. ^44^).

### HcMel12 transduction

The catalytic mutant of cyto*Lb*NOX was designed and the open reading frame was synthesized with mutations in Asp177 and Phe422 to alanine. Both wild-type and mutant open reading frames were cloned into the lentiviral plasmid pLex303 through the *NheI* and *BamHI* restriction sites, and transduction of HcMel12 was performed as previously described^50^. Transduced cells were selected by supplementation of 8 µg m^−1^ blasticidin, and single clones were selected from the surviving bulk population. cyto*Lb*NOX and catalytic mutant expression was confirmed using immunoblotting against the FLAG epitope. pLEX303 was a gift from D. Bryant (Addgene, plasmid no. 162032; http://n2t.net/addgene:162032; RRID: Addgene_162032).

### Hartwig dataset analysis

The HMF dataset included whole-genome sequencing data from tumor metastases normal-matched samples from 355 patients with melanoma (skin primary tumor location), of whom 233 had additional RNA sequencing data of the tumor samples. mtDNA somatic mutations were called and annotated as previously described^3,4^. In brief, variants called by both Mutect2 and SAMtools mpileup were retained and merged using vcf2maf, which embeds the variant effect predictor variant annotator. Variants within the repeat regions (chrM:302–315, chrM:513–525, and chrM:3105–3109) were filtered out. Next, variants were filtered out if the VAF was lower than 1% in the tumor samples and lower than 0.24% in the normal sample, as previously described^4^. Finally, somatic variants were kept when supported by at least one read in both the forward and the reverse orientations. Samples with >50% VAF mtDNA complex I truncating (frameshift indels, translation start site and nonsense mutations) and missense mutations were classified as mutated, and the rest were classified as wild type. Gene expression data were obtained from the output generated by the isofox pipeline, provided by HMF. Adjusted transcript per million gene counts per sample were merged into a matrix. Gene expression and mutation data were used to perform differential expression analysis with DESeq2 in R using the DESeqDataSetFromMatrix function. GSEA was performed with fGSEA in R with a minimum set size of 15 genes, a maximum of 500 genes and 20,000 permutations, against the mSigDB Hallmark gene set collection (v.7.5.1). Normalized enrichment scores were ranked for significant upregulated and downregulated gene sets.

### Bone marrow-derived cells and splenocyte conditioning

#### Bone marrow-derived cell culture

Bone marrow was isolated from mouse tibias and femurs, and 2 × 106 cells were re-suspended in 7 ml of R10 (RPMI (Gibco)) containing 10% FBS (Gibco), 1% penicillin–streptomycin (Gibco), 1% l-glutamine (Gibco), 1% Hepes (Gibco), 1% non-essential amino acid solution (Gibco), 50 mM 2-mercaptoethanol (Thermo Scientific), 200 ng ml^−1^ FLT3L (made in-house) and 10 ng ml^−1^ recombinant murine granulocyte-macrophage colony-stimulating factor (PeproTech) and incubated at 37 °C and 5% CO_2_. After 3 days, 10 ml of fresh media was added to each well, and cells were re-suspended in fresh media on day 6.

#### Splenocyte cell culture

Spleens were mashed through a 70 µM cell strainer, using FACS buffer to wash through all cells. Samples were then pelleted at 800×*g* for 2 min, then re-suspended in red blood cell lysis (RBCL) buffer (water containing 0.2 M ammonium chloride, 12 mM potassium bicarbonate and 0.01% EDTA) and incubated at room temperature (20 °C) for 10 min. Samples were pelleted and the RBCL incubation was repeated until the samples were no longer red. Cells were then re-suspended in R10 containing 5 µg ml^−1^ recombinant murine IL-2 (Peprotech) and incubated at 37 °C and 5% CO_2_ overnight.

#### Cell conditioning

Bone marrow-derived cells and splenocyte cultures were re-suspended in either conditioned media, un-conditioned media or media supplemented with 1 ng ml^−1^ of IFNγ. Samples were incubated at 37 °C and 5% CO_2_ overnight. Samples were then pelleted and used either for immunoblotting or flow cytometry. Immunoblotting was performed as described in the Methods using STAT1 antibody (10144-2-AP) and Phospho-STAT1 (Tyr701) antibody (28979-1-AP). Blots were quantified using Image Studio Lite (Li-Cor, v.5.2).

### Flow cytometry

#### Tumor processing

Tumors were dissected and chopped into small pieces and then re-suspended in digestion buffer (RPMI (Gibco)) containing 100 U ml^−1^ Collagenase IV (Sigma), 500 U ml^−1^ Collagenase II (Sigma) and 0.2 mg ml^−1^ DNase I (Sigma)). Samples were incubated on a shaker at 37 °C for 40 min and vigorously shaken at the 20 and 40 min mark.

#### Lymph node processing

Lymph nodes were split using tweezers and re-suspended in digestion buffer. Samples were incubated at 37 °C for 40 min. Samples were re-suspended in FACS buffer (PBS containing 2% FBS (Gibco)) and transferred into round-bottomed 96-well plates. Tumor and lymph node samples were passed through a 70 µM cell strainer (Fisher Scientific) and pelleted at 800×*g* for 2 min. Samples were re-suspended in FACS buffer and transferred into round-bottomed 96-well plates and pelleted.

#### Spleen processing

Spleens were mashed through a 70 µM cell strainer, using FACS buffer to wash through all cells. Samples were then pelleted at 800×*g* for 2 min and then re-suspended in FACS buffer and transferred into round-bottomed 96-well plates and re-pelleted. Samples were re-suspended in 200 µl RBCL buffer (water containing 0.2 M ammonium chloride, 12 mM potassium bicarbonate and 0.01% EDTA) and incubated at room temperature for 10 min. Samples were pelleted and the RBCL incubation was repeated until the samples were no longer red.

#### Sample staining and fixing

Samples were re-suspended in FACS buffer (PBS containing 2% FBS (Gibco)) + 1:1,000 Zombie NIR stain (BioLegend) and plated onto 96-well round-bottomed plates. Samples were incubated at 4 °C for 30 min, then pelleted and re-suspended in 200 µl of 1:400 panel antibodies + TruStain FcX (BioLegend). Plates were incubated at 4 °C for at least 40 min. After pelleting, samples were re-suspended in 200 µl 4% Pierce 16% formaldehyde (w/v) (Life Technologies) and incubated at room temperature for 10 min in the dark. Samples were pelleted and re-suspended in FACS buffer, sealed in parafilm and stored at 4 °C until analyzed.

To run the samples, each well was transferred into 1.2 ml round-bottom tubes (Stellar Scientific) and run on the BD LSRFortessa (BD Biosciences). Samples were analyzed using FlowJo (v.10.9.0).

### Statistics and reproducibility

No statistical test was used to determine sample sizes. Mice were randomly assigned to different experimental groups. Mice that were killed before the defined endpoint owing to ulceration were not included. Samples were blinded to machine operators (metabolomics, proteomics, RNA-seq). Researchers were blinded to experimental groups for in vivo ICB experiments. Samples for metabolomics were randomized by operators. Samples for metabolomics, proteomics and RNA-seq were blinded to the operators. All remaining data collection and analysis were not performed blind to the conditions of the experiments. Data distribution was assumed to be normal but this was not formally tested. Data outliers, determined using the Grubbs’ test, were removed before statistical testing. Specific statistical tests used to determine significance, group sizes (*n*) and *P* values are provided in the figure legends. Unless specified, experiments were conducted to have three biological replicates, each with three technical replicates, to ensure reproducibility and allow for accurate statistical measurements. No power calculations were performed; rather, sample sizes were chosen based on standards in the field for similar experiments. Asterisks indicate significant *P* values in figures, represented as *P < 0.05, **P < 0.01, ***P < 0.001 and ****P < 0.0001. All statistical analyses were carried out using Prism (v.9; GraphPad) and RStudio (v.2022.07.0). Complete figures were then constructed in Adobe Illustrator (v.2023; Adobe).

### Reporting summary

Further information on research design is available in the Nature Portfolio Reporting Summary linked to this article.

### Supplementary information

Supplementary Information PCR primer sequences (Supplementary Table 1).

Reporting Summary

### Source data

Source Data Fig. 1 Unprocessed western Blots and/or gels. Source Data Fig. 4 Unprocessed tumor images. Source Data Extended Data Fig. 1 Unprocessed western Blots and/or gels. Source Data Extended Data Fig. 2 Unprocessed western Blots and/or gels. Source Data Extended Data Fig. 3 Unprocessed tumor H&E stains. Source Data Extended Data Fig. 5 Unprocessed western Blots and/or gels. Source Data Extended Data Fig. 7 Unprocessed western Blots and/or gels. Source Data Extended Data Fig. 9 Unprocessed western Blots and/or gels.

Source Data Fig. 1–4 and Source Data Extended Data Fig. 1–7, 9, 10 Figure source data

## Data Availability

All reagents used in this study are either commercially available or can be made available from the corresponding authors upon reasonable request. All metabolomic data were uploaded to MassIVE (MSV000091475), mtDNA sequencing, bulk and single-cell RNA-seq were uploaded to GEO (GSE230677 and GSE227467) and proteomic data were uploaded to PRIDE (PXD044987 and PXD039705). All other data are available in the Supplementary information in the specified public repositories. Source data are provided with this paper.

## Code availability

All custom code is available through the Reznik lab Github (https://github.com/reznik-lab).
