# Supplementary material for: Mitochondrial DNA mutations drive aerobic glycolysis to enhance checkpoint blockade response in melanoma
Source: Nat Cancer. 2024 Jan 29;5(4):659–72. doi: 10.1038/s43018-023-00721-w (PMC11056318; doi:10.1038/s43018-023-00721-w)
Supplement: Supplementary file 1 — PCR primer sequences (Supplementary Table 1). [file 43018_2023_721_MOESM1_ESM.pdf]

nature cancer

Article https://doi.org/10.1038/s43018-023-00721-w

Mitochondrial DNA mutations drive aerobic glycolysis to enhance checkpoint blockade response in melanoma

In the format provided by the authors and unedited

Supplementary Table 1. PCR primer sequences.

Use	Orientation	Sequence
Long Range PCR	Forward 1	5’-ACTGATATTACTATCCCTAGGAGG-3’
Long Range PCR	Reverse 1	5’-TTTGAGTAGAACCCTGTTAGG-3’
Long Range PCR	Forward 2	5’-GGCCTGATAATAGTGACGC-3’
Long Range PCR	Reverse 2	5’-GGTTGGGTTTAGTTTTTGTTTGG-3’
PyroMark PCR of m.12,436	Forward	5’-ATATTCTCCAACAACAACG-3’
PyroMark PCR of m.12,436	Reverse	5’-**biotin**-GTTATTATTAGTCGTGAGG-3’
PyroMark PCR of m.12,436	Sequencing	5’-TTGGCCTCCACCCAT-3’
PyroMark PCR of m.11,944	Forward	5’-CTTCATTATTAGCCTCTTAC-3’
PyroMark PCR of m.11,944	Reverse	5’-**biotin**-GTCTGAGTGTATATATCATG-3’
PyroMark PCR of m.11,944	Sequencing	5’-TAATTACAACCTGGCACT-3’
ddPCR - mt-Nd5	Forward	5’-TGCCTAGTAATCGGAAGCCTCGC-3’
ddPCR - mt-Nd5	Reverse	5’-TCAGGCGTTGGTGTTGCAGG-3’
ddPCR – VDAC1	Forward	5’-CTCCCACATACGCCGATCTT-3’
ddPCR – VDAC1	Reverse	5’-GCCGTAGCCCTTGGTGAAG-3’
LbNOX Cloning	Forward	5’-GGTGGTGCTAGCCGCATGAAGGTCACCG-3’
mitoLbNOX Cloning	Forward	5’-GGTGGTGCTAGCCGCATGCTCGCTACAAG-3’
LbNOX Cloning	Reverse	5’-GGTGGTGGATCCTTACTTGTCATCGTCATC-3’
