# Supplementary material for: Mitochondrial DNA mutations drive aerobic glycolysis to enhance checkpoint blockade response in melanoma
Source: Nat Cancer. 2024 Jan 29;5(4):659–72. doi: 10.1038/s43018-023-00721-w (PMC11056318; doi:10.1038/s43018-023-00721-w)
Supplement: Supplementary file 2 — Reporting Summary [file 43018_2023_721_MOESM2_ESM.pdf]

nature portfolio

Corresponding author(s): Payam Gammage and Ed Reznik

Last updated by author(s): Nov 7, 2023

Reporting Summary

Nature Portfolio wishes to improve the reproducibility of the work that we publish. This form provides structure for consistency and transparency in reporting. For further information on Nature Portfolio policies, see our Editorial Policies and the Editorial Policy Checklist.

Statistics

For all statistical analyses, confirm that the following items are present in the figure legend, table legend, main text, or Methods section.

n/a	Confirmed	
☐	☒	The exact sample size (*n*) for each experimental group/condition, given as a discrete number and unit of measurement
☐	☒	A statement on whether measurements were taken from distinct samples or whether the same sample was measured repeatedly
☐	☒	The statistical test(s) used AND whether they are one- or two-sided<br>*Only common tests should be described solely by name; describe more complex techniques in the Methods section.*
☒	☐	A description of all covariates tested
☒	☐	A description of any assumptions or corrections, such as tests of normality and adjustment for multiple comparisons
☐	☒	A full description of the statistical parameters including central tendency (e.g. means) or other basic estimates (e.g. regression coefficient) AND variation (e.g. standard deviation) or associated estimates of uncertainty (e.g. confidence intervals)
☐	☒	For null hypothesis testing, the test statistic (e.g. *F*, *t*, *r*) with confidence intervals, effect sizes, degrees of freedom and *P* value noted<br>*Give P values as exact values whenever suitable.*
☒	☐	For Bayesian analysis, information on the choice of priors and Markov chain Monte Carlo settings
☒	☐	For hierarchical and complex designs, identification of the appropriate level for tests and full reporting of outcomes
☐	☒	Estimates of effect sizes (e.g. Cohen's *d*, Pearson's *r*), indicating how they were calculated

Our web collection on statistics for biologists contains articles on many of the points above.

Software and code

Policy information about availability of computer code

Data collection	Western blots were imaged using the Li-Cor Odyssey CLx with ImageStudio (v.5.2).<br>ddPCR data was collected using the BioRad digital droplet PCR system with QX Manager Software Standard Edition (v.2.1).<br>Metabolite abundance was measured using the Q Exactive Orbitrap mass spectrometer (ThermoFisher) coupled to an Ultimate 3000 HPLC system (ThemoFisher).<br>Proteomics was performed using the EASY-nLC II 1200 (ThermoFisher) coupled to an Orbitrap Fusion Lumos mass spectrometer (ThermoFisher) as part of nanoscale C18 reverse-phase liquid chromatography.<br>Basal oxygen consumption was measuring using the Seahorse XF Analyzer (Agilent).

Data analysis

Figure panels for numerical data were created using Prism (GraphPad, v9) and Rstudio (v.2022.07.0). Complete figures were then constructed in Adobe Illustrator (Adobe, v.2023).
Flow cytometry data was analysed using FlowJo (v.10.9.0).
Metabolomics data was analysed using Tracefinder (ThermoFisher, v.5.1).
Bulk transcriptomics data was analysed using STAR 2-pass alignment (v.2.7.10a), the edgeR package (v.3.40.1), limma package (v.3.54.0), fgsea R package (v.1.22.0) on R (v.4.2.1). Single-cell transcriptomics data was analysed using CellRanger (v.7.0.1), Seurat (v.4.2.0) package the clustifyr R package (v.1.8.0), clustifyrdatahub (v.1.6.0) and CopyKat (v.1.1.0) on R (v.4.2.1). Code can be accessed at: https://github.com/reznik-lab/engineered-mtDNA-mutation-in-tumor.
Human data was analysed using R (v.4.2.1).
Proteomics data was analysed using MaxQuant software (v.1.6.1.4) and Perseus software (v.1.6.13.0).
Western blots were quantified using Image Studio Lite (Li-Cor, v.5.2)
Proliferation data was analysed using the Incucyte Zoom 2018A software.
H&E slides were analysed using Aperio ImageScope (v.12.4.6.5003).

For manuscripts utilizing custom algorithms or software that are central to the research but not yet described in published literature, software must be made available to editors and reviewers. We strongly encourage code deposition in a community repository (e.g. GitHub). See the Nature Portfolio guidelines for submitting code & software for further information.

Data

Policy information about availability of data

All manuscripts must include a data availability statement. This statement should provide the following information, where applicable:

- Accession codes, unique identifiers, or web links for publicly available datasets
- A description of any restrictions on data availability
- For clinical datasets or third party data, please ensure that the statement adheres to our policy

All non-commercial plasmids used have been deposited with addgene (Gammage Lab). All metabolomic data were uploaded to MassIVE (MSV000091475), mtDNA sequencing, bulk and single cell RNAseq were uploaded to GEO (GSE230677 and GSE227467) and proteomic data were uploaded to PRIDE (PXD044987 and PXD039705). All other data are available in the supplementary information or via specified public repositories.

Research involving human participants, their data, or biological material

Policy information about studies with human participants or human data. See also policy information about sex, gender (identity/presentation), and sexual orientation and race, ethnicity and racism.

Reporting on sex and gender	Human data within this manuscript was accessed through publicly available datasets. We did not perform any human experiments ourselves.
Reporting on race, ethnicity, or other socially relevant groupings	N/A
Population characteristics	N/A
Recruitment	N/A
Ethics oversight	N/A

Note that full information on the approval of the study protocol must also be provided in the manuscript.

Field-specific reporting

Please select the one below that is the best fit for your research. If you are not sure, read the appropriate sections before making your selection.

☒ Life sciences ☐ Behavioural & social sciences ☐ Ecological, evolutionary & environmental sciences

For a reference copy of the document with all sections, see nature.com/documents/nr-reporting-summary-flat.pdf

Life sciences study design

All studies must disclose on these points even when the disclosure is negative.

Sample size	No power calculations were performed. Sample sizes were chosen based on standards in the field for similar experiments.
Data exclusions	Outliers, as recognized using the Grubbs' test, were excluded from datasets.<br>4434 and B78 tumours are prone to ulceration and these were excluded from downstream analyses.
Replication	All experiments were repeated to have at least three biological replicates, each with three technical replicated unless stated otherwise in figure legends.

Randomization	Samples for metabolomics were randomized by operators. Unless otherwise stated, no other samples within an experiment were randomized as they were not applicable to the experimental design.
Blinding	Samples for metabolomics, proteomics and RNAseq were blinded to the operators. Immune checkpoint inhibitors were blinded to the drug administrator. Unless otherwise stated, all other experiments were not blinded due to sample preparation and execution being conducted by a single experimenter.

Reporting for specific materials, systems and methods

We require information from authors about some types of materials, experimental systems and methods used in many studies. Here, indicate whether each material, system or method listed is relevant to your study. If you are not sure if a list item applies to your research, read the appropriate section before selecting a response.

Materials & experimental systems

n/a	Involved in the study
☐	☒ Antibodies
☐	☒ Eukaryotic cell lines
☒	☐ Palaeontology and archaeology
☐	☒ Animals and other organisms
☒	☐ Clinical data
☒	☐ Dual use research of concern
☒	☐ Plants

Methods

n/a	Involved in the study
☒	☐ ChIP-seq
☐	☒ Flow cytometry
☒	☐ MRI-based neuroimaging

Antibodies

Antibodies used

Mouse dosing:
Ultra-LEAF™ Purified anti-mouse CD279 (PD-1), Clone - RMP1-14, diluted to 100mg/mL (Biolegend #114122)
InVivoPlus Mouse, anti-Mouse CTLA-4 (CD152), Clone - 9D9 diluted to 100mg/mL (2bScientific, #BP0164)
InVivoPlus Rat, anti-Mouse Ly6G, Clone - 1A8 diluted to 100mg/mL (2Bscientific, #BP0075-1)
Ultra-LEAF™ Purified anti-mouse CD274 (B7-H1, PD-L1) Antibody, Clone – 10F.9G2, diluted to 100mg/mL (BioLegend, #124339)
Western blotting:
Anti-HA High Affinity from rat IgG1, Clone – 3F10, diluted 1:1000 (Roche, #11867423001)
Monoclonal ANTI-FLAG® Clone M2 antibody produced in mouse diluted 1:1000 (Sigma, #F1804)
anti-beta actin antibody, Clone mAbcam 8226, diluted 1:10,000 (Abcam, #ab8226)
MDH1 Monoclonal antibody diluted 1:1000 (Proteintech, #66505-1-Ig)
Total OXPHOS Rodent WB Antibody Cocktail, Clone – 1F9A2, diluted 1:800 (Abcam, #ab110413)
STAT1 antibody diluted 1:1000 (Proteintech, #10144-2-AP)
Phospho-STAT1 (Tyr701) antibody diluted 1:1000 (Proteintech, #28979-1-AP)
Recombinant Anti-Vinculin antibody, Clone – EPR8185, diluted 1:10,000 (Abcam, #ab129002)
IRDye® 800CW Donkey anti-Rabbit IgG Secondary Antibody (Li-Cor, #926-32213)
IRDye® 800CW Goat Anti-Mouse IgG Secondary Antibody (Li-Cor, #926-32210)
Flow cytometry:
Brilliant Violet 510™ anti-mouse CD11c, Clone - N418, Antibody diluted 1:400 (BioLegend, #117337)
Brilliant Violet 605™ anti-mouse CX3CR1, Clone - YE1/19.1, Antibody diluted 1:400 (BioLegend, #149207)
Brilliant Violet 711™ anti-mouse F4/80, Clone - BM8, Antibody diluted 1:400 (BioLegend, #123147)
FITC anti-mouse/human CD11b Antibody, Clone - M1/70, diluted 1:400 (BioLegend, #101205)
PerCP/Cyanine5.5 anti-mouse Ly-6C, Clone - HK1.4, Antibody diluted 1:400 (BioLegend, #128011)
Alexa Fluor® 647 anti-mouse I-A/I-E Antibody, Clone - M5/114.15.2, diluted 1:400 (BioLegend, 107617)
APC anti-mouse Ly-6G Antibody diluted 1:400, Clone - 1A8, (BioLegend, #127613)
PE anti-mouse CD80, Clone - 16-10A1, Antibody diluted 1:400 (BioLegend, #104707)
Alexa Fluor® 700 anti-mouse CD45, Clone - 30-F11, Antibody diluted 1:400 (BioLegend, #103127)
Brilliant Violet 421™ anti-mouse CD45, Clone - 30-F11, Antibody diluted 1:400 (BioLegend, #103133)
Brilliant Violet 605™ anti-mouse CD3, Clone - 17A2, Antibody diluted 1:400 (BioLegend, #100237)
Brilliant Violet 785™ anti-mouse/human CD45R/B220, Clone - RA3-6B2, Antibody diluted 1:400 (BioLegend, #103245)
FITC anti-mouse CD8a, Clone - 53-6.7, Antibody diluted 1:400 (BioLegend, #100705)
PerCP/Cyanine5.5 anti-mouse NK-1.1, Clone - PK136, Antibody diluted 1:400 (BioLegend, #108727)
PE anti-rat CD90/mouse CD90.1 (Thy-1.1), Clone - OX-7, Antibody diluted 1:400 (BioLegend, #202523)
PE/Cyanine7 anti-mouse CD4, Clone - RM4-5, Antibody diluted 1:400 (BioLegend, #100527)
Brilliant Violet 605™ anti-mouse/human CD11b, Clone - M1/70, Antibody diluted 1:400 (BioLegend, #101237)
Brilliant Violet 711™ anti-mouse CD45, Clone - 30-F11, Antibody diluted 1:400 (BioLegend, #103147)
FITC anti-mouse TER-119/Erythroid Cells, Clone - TER-119, Antibody diluted 1:400 (BioLegend, #116205)
FITC anti-mouse CD335 (NKp46), Clone - 29A1.4, Antibody diluted 1:400 (BioLegend, #137605)
CD115 (c-fms) Monoclonal Antibody FITC diluted 1:400 (Thermofisher, #AFS98)
FITC anti-mouse CD3, Clone - 17A2, Antibody diluted 1:400 (BioLegend, #100203)
FITC anti-mouse CD19, Clone 1D3/CD19, Antibody diluted 1:400 (BioLegend, #152403)
Alexa Fluor® 647 anti-mouse CD170 (Siglec-F), Clone - S17007L, Antibody diluted 1:400 (BioLegend, #155519)
PE/Cyanine7 anti-mouse Ly-6G, Clone - 1A8, Antibody diluted 1:400 (BioLegend, #127617)
Brilliant Violet 510™ anti-mouse F4/80, Clone - BM8, Antibody diluted 1:400 (BioLegend, #123135)

Brilliant Violet 650™ anti-mouse CD11c, Clone - N418, Antibody diluted 1:400 (BioLegend, #117339)
Brilliant Violet 711™ anti-mouse NK-1.1, Clone - PK136, Antibody diluted 1:400 (BioLegend, 108745)
PE anti-mouse Ly-6G, Clone - 1A8, Antibody diluted 1:400 (BioLegend, #127607)
Alexa Fluor® 700 anti-mouse CD3, Clone - 17A2, Antibody diluted 1:400 (BioLegend, #100215)
All antibodies were used as per the manufacturer's instructions.

Validation

These antibodies are widely used commercially, and were validated in our usage through inclusion of standard controls in our experiments.
Mouse dosing:
Ultra-LEAF™ Purified anti-mouse CD279 (PD-1), Clone - RMP1-14, diluted to 100mg/mL (Biolegend #114122): validated by manufacturer and used in peer-reviewed articles eg.: 1- Kanai, T., et al. 2003. J. Immunol. (FC, IHC), Yamazaki, T., et al. 2005. J. Immunol. (Costim), Matsumoto, K., et al. 2004. J. Immunol. (Block)
InVivoPlus Mouse, anti-Mouse CTLA-4 (CD152), Clone - 9D9 diluted to 100mg/mL (2bScientific, #BP0164): validated by manufacturer and used in peer-reviewed articles eg: 1- Kleczko, E. K., Nguyen, D. T., et al. 2023. JCI Insight., 2- Chen, Y., Sun, J., et al. 2022. J. of Trans. Med., 3- Potluri, H. K., Ferreira, C. A., et al. 2022. J. for Immunotherapy of Cancer.
InVivoPlus Rat, anti-Mouse Ly6G, Clone - 1A8 diluted to 100mg/mL (2Bscientific, #BP0075-1): validated by manufacturer and used in peer-reviewed articles eg: 1- Cao, L., Ma, L., et al. 2023. eLife, 2- Gullotta, G. S., De Feo, D., et al. 2023. Nat. Imm., 3- Gong, H. H., Worley, M. J., et al. 2023. Front. in Imm.
Ultra-LEAF™ Purified anti-mouse CD274 (B7-H1, PD-L1) Antibody, Clone – 10F.9G2, diluted to 100mg/mL (BioLegend, #124339): validated by manufacturer and used in peer-reviewed articles eg: 1- Maier H, et al. 2007. J. Immunol., 2- Scarlett UK, et al. 2012. J Exp Med., 3- Paterson AM, et al. 2011. J. Immunol.
Western blotting:
Anti-HA High Affinity from rat IgG1, Clone – 3F10, diluted 1:1000 (Roche, #11867423001): validated by manufacturer and used in peer-reviewed articles eg: 1- Halbleib et al. 2017. Molecular cell., 2- Nissanka et al. 2018. Nature Comm., 3- Amodeo et al. 2018. J. of Cell Sci.
Monoclonal ANTI-FLAG® Clone M2 antibody produced in mouse diluted 1:1000 (Sigma, #F1804): validated by manufacturer and used in peer-reviewed articles eg: 1- Yumin Qiu et al. 2019. Mol. Cell. Res., 2- Yan Han et al. 2010. J. of Bio. Chem. 3- Srivastava et al. 2015. Nat. Comm.
anti-beta actin antibody, Clone mAbcam 8226, diluted 1:10,000 (Abcam, #ab8226): validated by manufacturer and used in peer-reviewed articles eg: 1- Wang et al. 2023. Histol. Histopathol., 2- Liu et al. 2023. Cell Biochem., 3- Zheng et al. 2023. Cancer Gene Ther.
MDH1 Monoclonal antibody diluted 1:1000 (Proteintech, #66505-1-Ig)
Total OXPHOS Rodent WB Antibody Cocktail, Clone – 1F9A2, diluted 1:800 (Abcam, #ab110413)
STAT1 antibody diluted 1:1000 (Proteintech, #10144-2-AP): validated by western blotting by manufacturer using A431 cells, HEK-293 cells, A549 cells, K-562 cells, PC-3 cells.
Phospho-STAT1 (Tyr701) antibody diluted 1:1000 (Proteintech, #28979-1-AP): validated by western blotting by manufacturer using IFN gamma and LPS treated THP-1 cells.
Recombinant Anti-Vinculin antibody, Clone – EPR8185, diluted 1:10,000 (Abcam, #ab129002): validated by manufacturer and used in peer-reviewed articles eg: 1- Drazic et al. 2022. J. Mol. Bio., 2- Cheng et al. 2022. Cancer Comm., 3- Duran et al. 2022. J. Bone. Miner.
IRDye® 800CW Donkey anti-Rabbit IgG Secondary Antibody (Li-Cor, #926-32213)
IRDye® 800CW Goat Anti-Mouse IgG Secondary Antibody (Li-Cor, #926-32210)
Flow cytometry:
Brilliant Violet 510™ anti-mouse CD11c, Clone - N418, Antibody diluted 1:400 (BioLegend, #117337)): validated by manufacturer and used in peer-reviewed articles eg: 1- Granucci F, et al. 1997. J. Immunol., 2- Wikstrom M, et al.2006. J. Immunol., 3- Ramakrishna C, et al. 2019. Nat Commun.
Brilliant Violet 605™ anti-mouse CX3CR1, Clone - YE1/19.1, Antibody diluted 1:400 (BioLegend, #149207): validated by manufacturer and used in peer-reviewed articles eg: 1- Wang H, et al. 2012. Proc Natl Acad Sci USA, 2- Wolock SL, et al. 2019. Cell Rep.
Brilliant Violet 711™ anti-mouse F4/80, Clone - BM8, Antibody diluted 1:400 (BioLegend, #123147): validated by manufacturer and used in peer-reviewed articles eg: 1- Schaller E, et al. 2002. Mol. Cell. Biol., 2- Kathryn A Pape et al. 2018. Immunity., 3- Chow MT et al. 2019. Immunity.
FITC anti-mouse/human CD11b Antibody, Clone - M1/70, diluted 1:400 (BioLegend, #101205): validated by manufacturer and used in peer-reviewed articles eg: 1- Narusawa M, et al. 2014. Cancer Immunol, Ostapoff K, et al. 2014. Cancer Res., Fan X, et al. 2015. Cancer Res.
PerCP/Cyanine5.5 anti-mouse Ly-6C, Clone - HK1.4, Antibody diluted 1:400 (BioLegend, #128011): validated by manufacturer and used in peer-reviewed articles eg: 1- Ballesteros I, et al. 2014. J Vis Exp., 2- Gallizioli M, et al. 2020. Cell Rep., 3- Lin C et al. 2019. Immunity.
Alexa Fluor® 647 anti-mouse I-A/I-E Antibody, Clone - M5/114.15.2, diluted 1:400 (BioLegend, 107617): validated by manufacturer and used in peer-reviewed articles eg: 1- Hu HJ, et al. 2020. Cell Death Dis., 2- Shen Y, et al. 2021. Comput Struct Biotechnol J., 3- Mercer HL, et al. 2020. PLoS Pathog.
APC anti-mouse Ly-6G Antibody diluted 1:400, Clone - 1A8, (BioLegend, #127613): validated by manufacturer and used in peer-reviewed articles eg: 1- Xue F, et al. 2020. Int J Biol Sci., 2- Patras KA, et al. 2019. J Innate Immun., 3- Jones NM, et al. 2019. BMC Cancer.
PE anti-mouse CD80, Clone - 16-10A1, Antibody diluted 1:400 (BioLegend, #104707): validated by manufacturer and used in peer-reviewed articles eg: 1- Koyama M, et al. 2015. J Exp Med., 2- Rozanski C, et al. 2011. J Exp Med., 3- Mulder R, et al. 2017. Front Immunol.
Alexa Fluor® 700 anti-mouse CD45, Clone - 30-F11, Antibody diluted 1:400 (BioLegend, #103127): validated by manufacturer and used in peer-reviewed articles eg: 1- Stoupa A, et al. 2018. EMBO Mol Med., 2- Komuczki J, et al. 2019. Immunity., 3- Saha D et al. 2017. Cancer Cell.
Brilliant Violet 421™ anti-mouse CD45, Clone - 30-F11, Antibody diluted 1:400 (BioLegend, #103133): validated by manufacturer and used in peer-reviewed articles eg: 1- Zahr A, et al. 2016. Nat Commun., 2- Schlecht A, et al. 2021. Int J Mol Sci., 3- Ulland TK et al. 2017. Cell.
Brilliant Violet 605™ anti-mouse CD3, Clone - 17A2, Antibody diluted 1:400 (BioLegend, #100237) validated by manufacturer and used in peer-reviewed articles eg: 1- Yu-Han Chang et al. 2017. Immunity., 2- Chandran S, et al. 2020. Front Immunol., 3- Aguilera T, et al. 2016. Nat Commun.
Brilliant Violet 785™ anti-mouse/human CD45R/B220, Clone - RA3-6B2, Antibody diluted 1:400 (BioLegend, #103245): validated by

manufacturer and used in peer-reviewed articles eg: 1- Ochiai S, et al. 2014. J Immunol., 2- Gallizioli M, et al. 2020. Cell Rep., 3- Rodda LB et al. 2018. Immunity.
FITC anti-mouse CD8a, Clone - 53-6.7, Antibody diluted 1:400 (BioLegend, #100705): validated by manufacturer and used in peer-reviewed articles eg: 1- Sun L, et al. 2021. Cancer Cell., 2- Logan K Smith et al. 2018. Immunity., 3- Strickley JD, et al. 2019. Nature.
PerCP/Cyanine5.5 anti-mouse NK-1.1, Clone - PK136, Antibody diluted 1:400 (BioLegend, #108727): validated by manufacturer and used in peer-reviewed articles eg: 1- Minute L, et al. 2020. J Immunother Cancer., 2- Kobayashi T, et al. 2019. Cell., 3- Trotta E, et al. 2018. Nat Med.
PE anti-rat CD90/mouse CD90.1 (Thy-1.1), Clone - OX-7, Antibody diluted 1:400 (BioLegend, #202523): validated by manufacturer and used in peer-reviewed articles eg: 1- Logan K Smith et al. 2018. Immunity., 2- Dong MB, et al. 2020. Cell., 3- Garber C, et al. 2019. Nat Neurosci.
PE/Cyanine7 anti-mouse CD4, Clone - RM4-5, Antibody diluted 1:400 (BioLegend, #100527): validated by manufacturer and used in peer-reviewed articles eg: 1- Quispe Calla N, et al. 2016. Sci Rep., 2- Kaczanowska S, et al. 2021. Cell., 3- Vanderleyden I, et al. 2020. Cell Rep.
Brilliant Violet 605™ anti-mouse/human CD11b, Clone - M1/70, Antibody diluted 1:400 (BioLegend, #101237): validated by manufacturer and used in peer-reviewed articles eg: 1- Gallizioli M, et al. 2020. Cell Rep., 2- Michela Miani et al. 2018. Cell Metabolism., 3- Komuczki J, et al. 2019. Immunity.
Brilliant Violet 711™ anti-mouse CD45, Clone - 30-F11, Antibody diluted 1:400 (BioLegend, #103147): validated by manufacturer and used in peer-reviewed articles eg: 1- Schloss MJ, et al. 2022. Nat Immunol., 2- Qi Z, et al. 2022. Nat Commun., 3- Harel M, et al. 2020. Cell.
FITC anti-mouse TER-119/Erythroid Cells, Clone - TER-119, Antibody diluted 1:400 (BioLegend, #116205): validated by manufacturer and used in peer-reviewed articles eg: 1- Suzuki M, et al. 2015. J Immunol., 2- Hou X, et al. 2020. Cell Reports., 3- Furuhashi K, et al. 2017. Immunology.
FITC anti-mouse CD335 (NKp46), Clone - 29A1.4, Antibody diluted 1:400 (BioLegend, #137605):): validated by manufacturer and used in peer-reviewed articles eg: 1-Calabrese DR, et al. 2020. J Clin Invest., 2-Huang J, et al. 2021. Immunity., 3-Shapiro MR, et al. 2020. Front Immunol.
CD115 (c-fms) Monoclonal Antibody FITC, Clone – AFS98, diluted 1:400 (Thermofisher, #AFS98): validated by manufacturer and used in peer-reviewed articles eg: 1- Lou et al. 2014. J. of Cell. Sci., 2-Hamilton et al. 2013. PLos One.
FITC anti-mouse CD3, Clone - 17A2, Antibody diluted 1:400 (BioLegend, #100203): validated by manufacturer and used in peer-reviewed articles eg: 1- Harsha Krovi S, et al. 2020. Nat Commun., 2- Dai L, et al. 2020. Cell., 3- Zaman R, et al. 2021. Immunity.
FITC anti-mouse CD19, Clone 1D3/CD19, Antibody diluted 1:400 (BioLegend, #152403): validated by manufacturer and used in peer-reviewed articles eg: 1- Chei S, et al. 2020. Front Nutr., 2- Zhou R, et al. 2022. EBioMedicine., Zhao J, et al. 2019. Nat Commun.
Alexa Fluor® 647 anti-mouse CD170 (Siglec-F), Clone - S17007L, Antibody diluted 1:400 (BioLegend, #155519): validated by manufacturer.
PE/Cyanine7 anti-mouse Ly-6G, Clone - 1A8, Antibody diluted 1:400 (BioLegend, #127617): validated by manufacturer and used in peer-reviewed articles eg: 1- Furuya Y, et al. 2014. J Virol., 2- Argüello RJ, et al. 2020. Cell Metab., 3- Bieren J, et al. 2015. J Immunol.
Brilliant Violet 510™ anti-mouse F4/80, Clone - BM8, Antibody diluted 1:400 (BioLegend, #123135): validated by manufacturer and used in peer-reviewed articles eg: 1- Lin YR, et al. 2020. Immunity., 2- Parks KR, et al. 2019. Cell Rep., 3- Linnerbauer M, et al. 2022. Front Immunol.
Brilliant Violet 650™ anti-mouse CD11c, Clone - N418, Antibody diluted 1:400 (BioLegend, #117339): validated by manufacturer and used in peer-reviewed articles eg: 1- Alkhani A, et al. 2020. Sci Rep., 2- Kaczanowska S, et al. 2021. Cell., 3- Barry KC, et al. 2018. Nat Med.
Brilliant Violet 711™ anti-mouse NK-1.1, Clone - PK136, Antibody diluted 1:400 (BioLegend, #108745): validated by manufacturer and used in peer-reviewed articles eg: 1- Komuczki J, et al. 2019. Immunity., 2- Kaczanowska S, et al. 2021. Cell., 3- Pokrovskii M, et al. 2019. Immunity.
PE anti-mouse Ly-6G, Clone - 1A8, Antibody diluted 1:400 (BioLegend, #127607): validated by manufacturer and used in peer-reviewed articles eg: 1- Lee T, et al. 2014. Mol Biol Cell., 2- DeSouza-Vieira T, et al. 2020. Cell Rep., 3- Bowling S, et al. 2020. Cell..
Alexa Fluor® 700 anti-mouse CD3, Clone - 17A2, Antibody diluted 1:400 (BioLegend, #100215): validated by manufacturer and used in peer-reviewed articles eg: 1- Hirai T, et al. 2020. Immunity., 2- Cignarella F et al. 2018. Cell Metabolism., 3- Roco JA et al. 2019. Immunity.

Eukaryotic cell lines

Policy information about cell lines and Sex and Gender in Research

Cell line source(s)	B78 (B78-D14) cells were sourced from ATCC. Hcmel12, YUMM1.7c7, YUMM1.7, 4434 and 5555 melanoma cells were gifted by Dr Amaya Viros (CRUK Manchester Institute)
Authentication	All cells were authenticated by in house authentication service through morphology and STR profiling.
Mycoplasma contamination	All cells were routinely checked for mycoplasma contamination and were negative.
Commonly misidentified lines (See ICLAC register)	No commonly misidentified cell lines were used in the study.

Animals and other research organisms

Policy information about studies involving animals; ARRIVE guidelines recommended for reporting animal research, and Sex and Gender in Research

Laboratory animals	C57/Bl6 male mice, purchased from Charles River, between 10 and 16 weeks of age were used for in vivo subcutaneous modelling of cancer.

Wild animals	No wild animals were used in this study.
Reporting on sex	Findings do not apply to one sex. Sex was not considered in this study.
Field-collected samples	No field-collected samples were used in this study.
Ethics oversight	Our research complies with all relevant ethical regulations. Animal experiments were carried out in accordance with the UK Animals (Scientific Procedures) Act 1986 (P72BA642F) and by adhering to the ARRIVE guidelines with approval from the local Animal Welfare and Ethical Review Board of the University of Glasgow. CRUK BI and UK Home Office ethical oversight guided experimental protocols, according to licensed procedures available.

Note that full information on the approval of the study protocol must also be provided in the manuscript.

Plants

Seed stocks	N/A
Novel plant genotypes	N/A
Authentication	N/A

Flow Cytometry

Plots

Confirm that:

- ☒ The axis labels state the marker and fluorochrome used (e.g. CD4-FITC).
- ☒ The axis scales are clearly visible. Include numbers along axes only for bottom left plot of group (a 'group' is an analysis of identical markers).
- ☒ All plots are contour plots with outliers or pseudocolor plots.
- ☒ A numerical value for number of cells or percentage (with statistics) is provided.

Methodology

Sample preparation	Tumours, spleens and lymph nodes were digested, filtered then stained with Zombie NIR Stain. After a 20min incubation at 4C, antibodies were added on at a 1:400 ratio in FACS buffer. Samples were incubated at 4C for 1hr then fixed and stored till run.
Instrument	BD LSRFortessa™ Cell Analyzer
Software	FlowJo v.10.9.0
Cell population abundance	Cells were not sorted into fractions. They were run on the Fortessa and sent straight to waste.
Gating strategy	Neutrophils ED Figure 23: FSC-A vs SSC-A for cells, FSC-A vs FSC-H for single cells, FSC-A vs Zombie (R780) for live cells, FSC-A vs CD45 (R730) for CD45+ cells, CD11b (B530) vs Ly6C (B685) to select for CD11b+ Ly6C+ cells, Ly6G (R670) vs Ly6C (B685) for Ly6G+ Ly6C+ neutrophils, mean fluorescence intensity (MFI) for CD11b (B530) was calculated from this population.<br>Monocytes ED Figure 23: FSC-A vs SSC-A for cells, FSC-A vs FSC-H for single cells, FSC-A vs Zombie (R780) for live cells, FSC-A vs CD45 (R730) for CD45+ cells, CD11b (B530) vs Ly6C (B685) to select for CD11b+ Ly6C+ cells, F4/80 (BV710) vs Ly6C (B685) for F4/80- Ly6C+ monocytes, MFI for CX3CR1 (BV605) was calculated from this population.<br>Macrophages ED Figure 23: FSC-A vs SSC-A for cells, FSC-A vs FSC-H for single cells, FSC-A vs Zombie (R780) for live cells, FSC-A vs CD45 (R730) for CD45+ cells, CD11b (B530) vs Ly6C (B685) to select for CD11b+ Ly6C- cells, CD11b (B530) vs F4/80 (BV710) for CD11b+ F4/80+ macrophages, MFI for MHCII (R670) was calculated from this population.<br>cDCs ED Figure 23: FSC-A vs SSC-A for cells, FSC-A vs FSC-H for single cells, FSC-A vs Zombie (R780) for live cells, FSC-A vs CD45 (R730) for CD45+ cells, Ly6C (B685) and F4/80 (BV710) vs FSC-A for Ly6C- and F4/80- cells, CD11c (BV510) vs MHCII (R670) for CD11c+ MHCII+ cDCs, MFI for MHCII (R670) and CD80 (YG586) was calculated from this population.<br>CD4+ T-cells Figure 23: FSC-A vs SSC-A for cells, FSC-A vs FSC-H for single cells, FSC-A vs Zombie (R780) for live cells, FSC-A vs CD45 (BV750) for CD45+ cells, CD3 (BV610) vs B220 (BV785) for CD3+ B220- cells, CD4 (YG780) vs CD8 (B530) for CD4+ T-cells, MFI for Thy1.1 (YG586) was calculated from this population.<br>CD8+ T-cells Figure 23: FSC-A vs SSC-A for cells, FSC-A vs FSC-H for single cells, FSC-A vs Zombie (R780) for live cells, FSC-A vs CD45 (BV750) for CD45+ cells, CD3 (BV610) vs B220 (BV785) for CD3+ B220- cells, CD4 (YG780) vs CD8 (B530) for CD8+ T-cells, MFI for Thy1.1 (YG586) was calculated from this population.<br>Neutrophils ED Figure 26: FSC-A vs SSC-A for cells, FSC-A vs FSC-H for single cells, FSC-A vs Zombie (R780) for live cells, FSC-A

vs CD45 (BV710) for CD45+ cells, FSC-A vs TER119, NKp46, CD115, CD3, CD19 (B530) to dump out other immune cells, SiglecF (R670) vs CD11b (BV610) to dump out SiglecF+ cells, CD11b (BV610) vs Ly6C (B685) to select for double positive neutrophils, Ly6C (B685) vs Ly6G (YG780) to clean up the previous population for double positive neutrophils.
CD4+ T-cells ED Figure 26: FSC-A vs SSC-A for cells, FSC-A vs FSC-H for single cells, FSC-A vs Zombie (R780) for live cells, FSC-A vs CD45 (BV450) for CD45+ cells, CD3 (R730) vs B220 (BV785) for CD3+ cells, CD4 (YG780) vs CD8 (B530) for CD4+ T-cells
CD4+ T-cells ED Figure 26: FSC-A vs SSC-A for cells, FSC-A vs FSC-H for single cells, FSC-A vs Zombie (R780) for live cells, FSC-A vs CD45 (BV450) for CD45+ cells, CD3 (R730) vs B220 (BV785) for CD3+ cells, CD4 (YG780) vs CD8 (B530) for CD8+ T-cells
NK T-cells ED Figure 26: FSC-A vs SSC-A for cells, FSC-A vs FSC-H for single cells, FSC-A vs Zombie (R780) for live cells, FSC-A vs CD45 (BV450) for CD45+ cells, CD3 (R730) vs B220 (BV785) for CD3- B220- cells, CD3 (R730) vs NK1.1 (B685) for CD3- NK1.1+ NK T-cells.
Macrophages ED Figure 26: FSC-A vs SSC-A for cells, FSC-A vs FSC-H for single cells, FSC-A vs Zombie (R780) for live cells, FSC-A vs CD45 (BV450) for CD45+ cells, CD11b (BV610) vs Ly6C (B685) for CD11b+ Ly6C- cells, CD11b (BV610) vs F4/80 (BV510) for CD11b+ F4/80+ macrophages.

☒ Tick this box to confirm that a figure exemplifying the gating strategy is provided in the Supplementary Information.
