# Supplementary material for: Mitochondrial DNA mutations drive aerobic glycolysis to enhance checkpoint blockade response in melanoma
Source: Nat Cancer. 2024 Jan 29;5(4):659–72. doi: 10.1038/s43018-023-00721-w (PMC11056318; doi:10.1038/s43018-023-00721-w)

Mahmood et al - Figure 1F

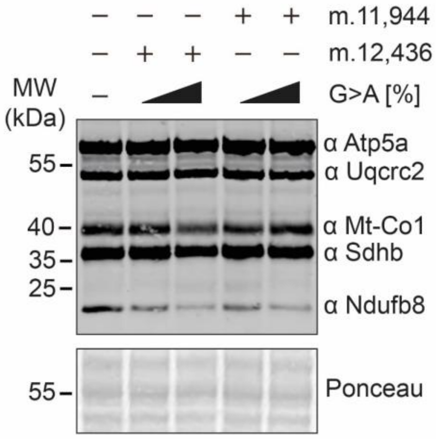

FIGURE BLOT

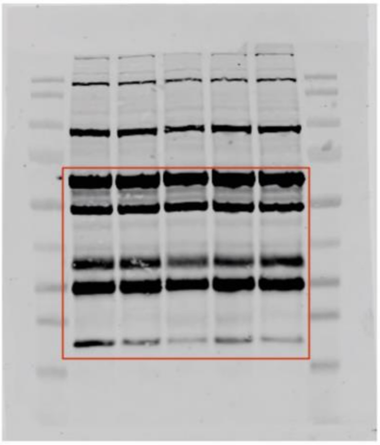

FIGURE PONCEAU

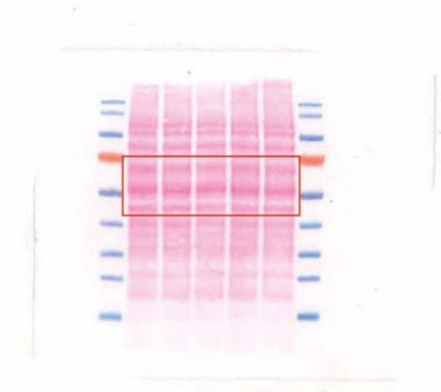

Mahmood et al - Figure 1G

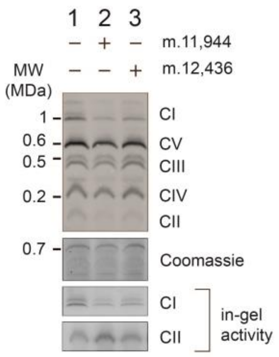

FIGURE OXPHOS

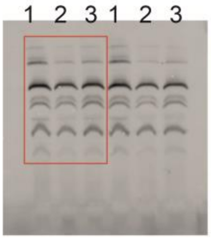

FIGURE COOMASSIE

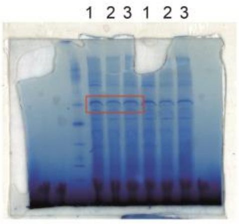

FIGURE COMPLEX I IGA

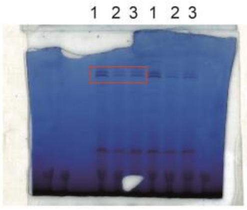

FIGURE COMPLEX II IGA

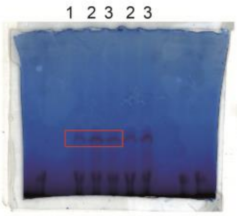

Mahmood et al - Figure 4B

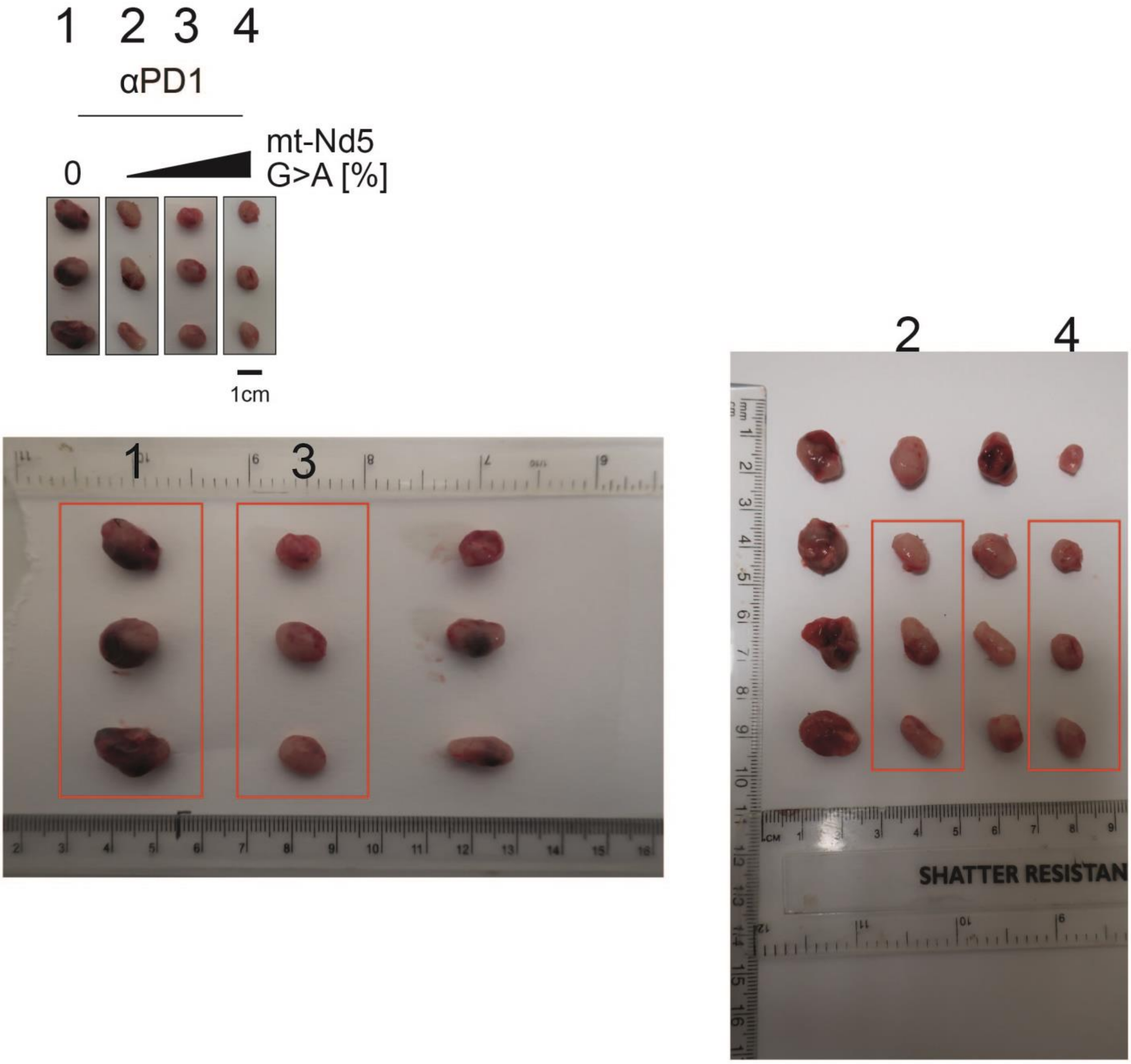

Mahmood et al - Figure 4E

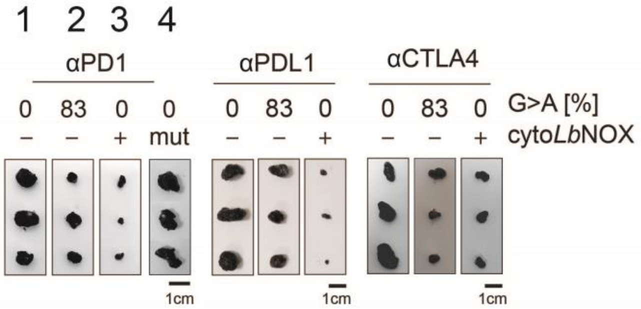

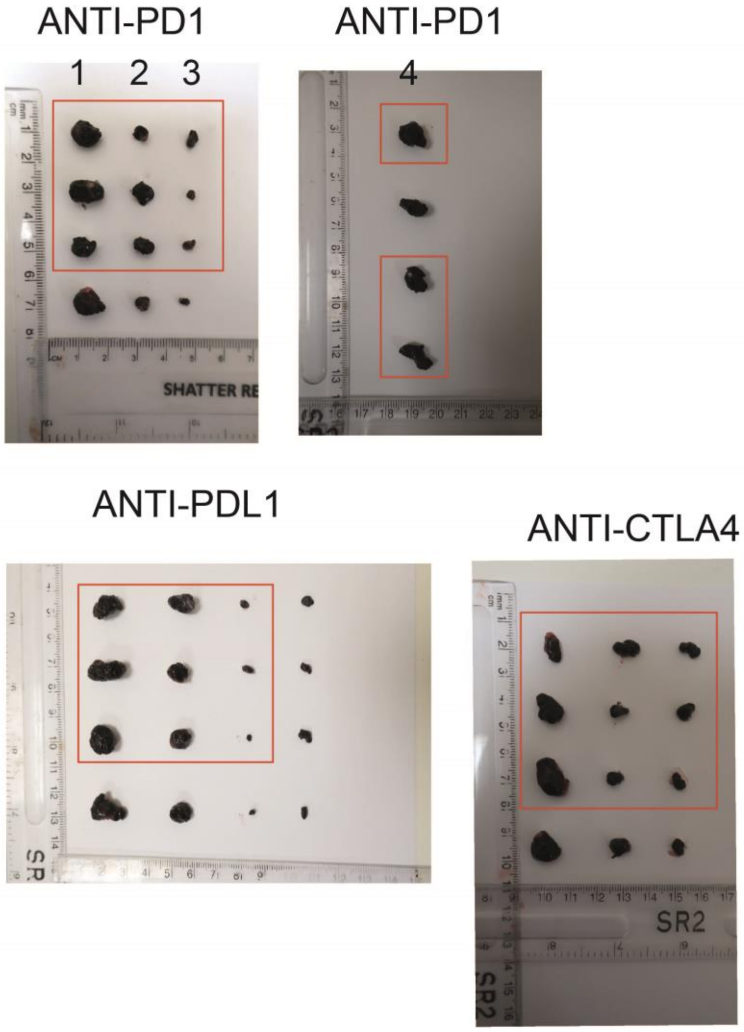

Mahmood et al - Figure 4H

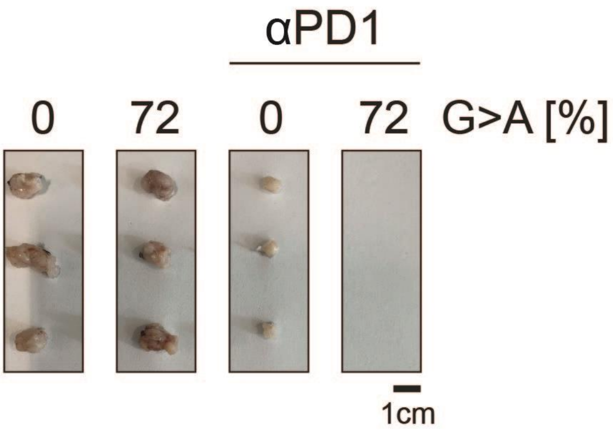

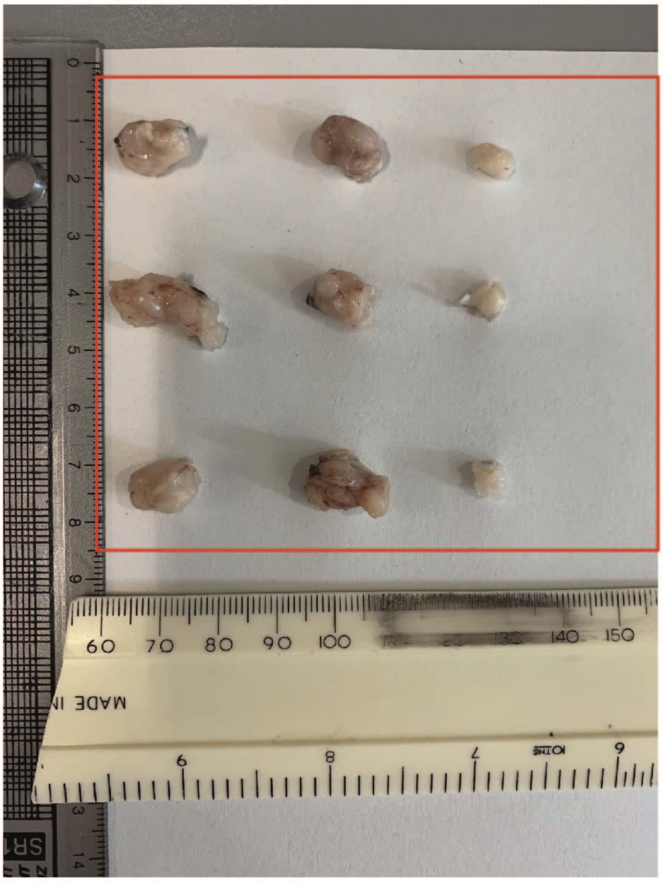

Mahmood et al - Extended Data Figure 1D

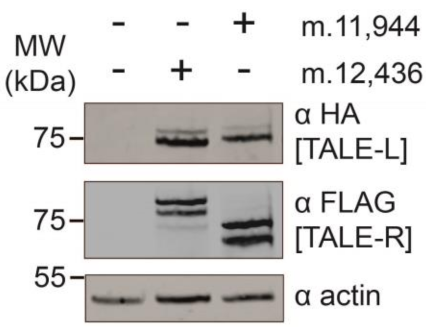

FIGURE HA

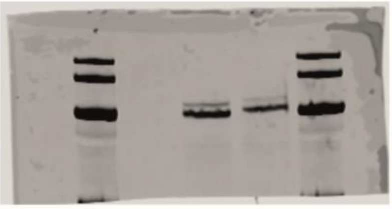

FIGURE FLAG

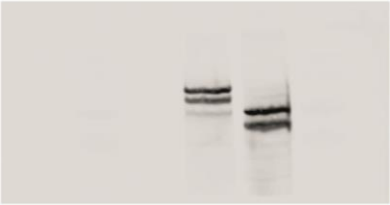

FIGURE ACTIN

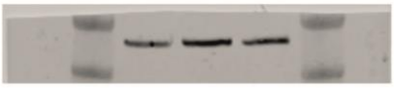

Mahmood et al - Extended Data Figure 1G

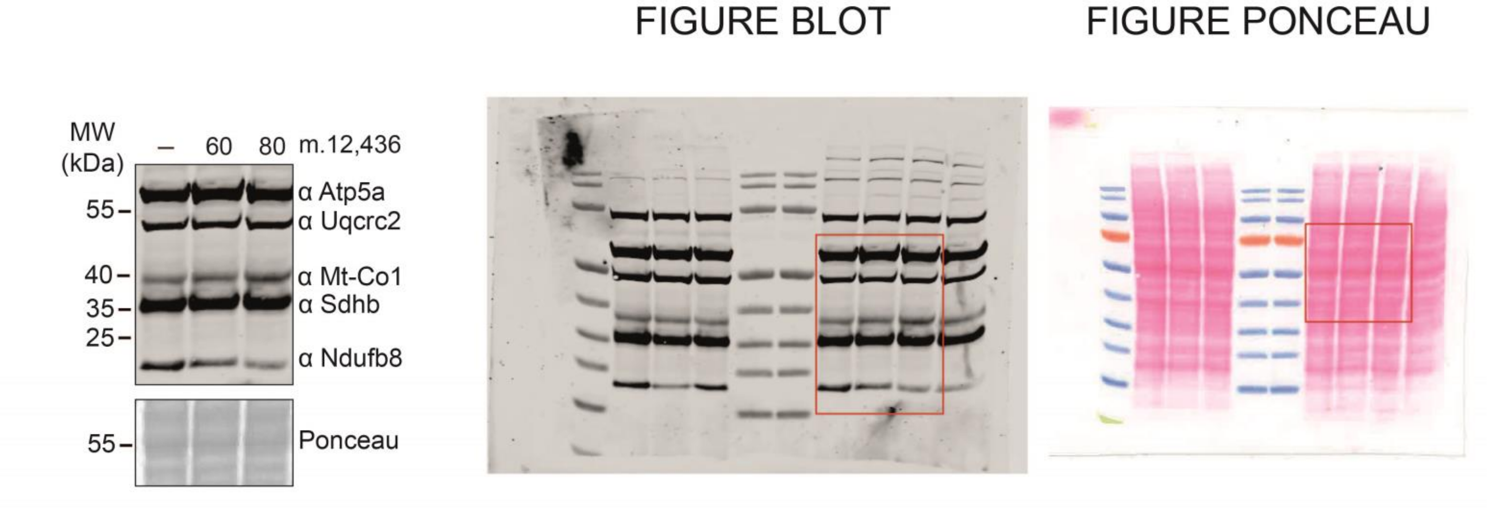

Mahmood et al - Extended Data Figure 2J

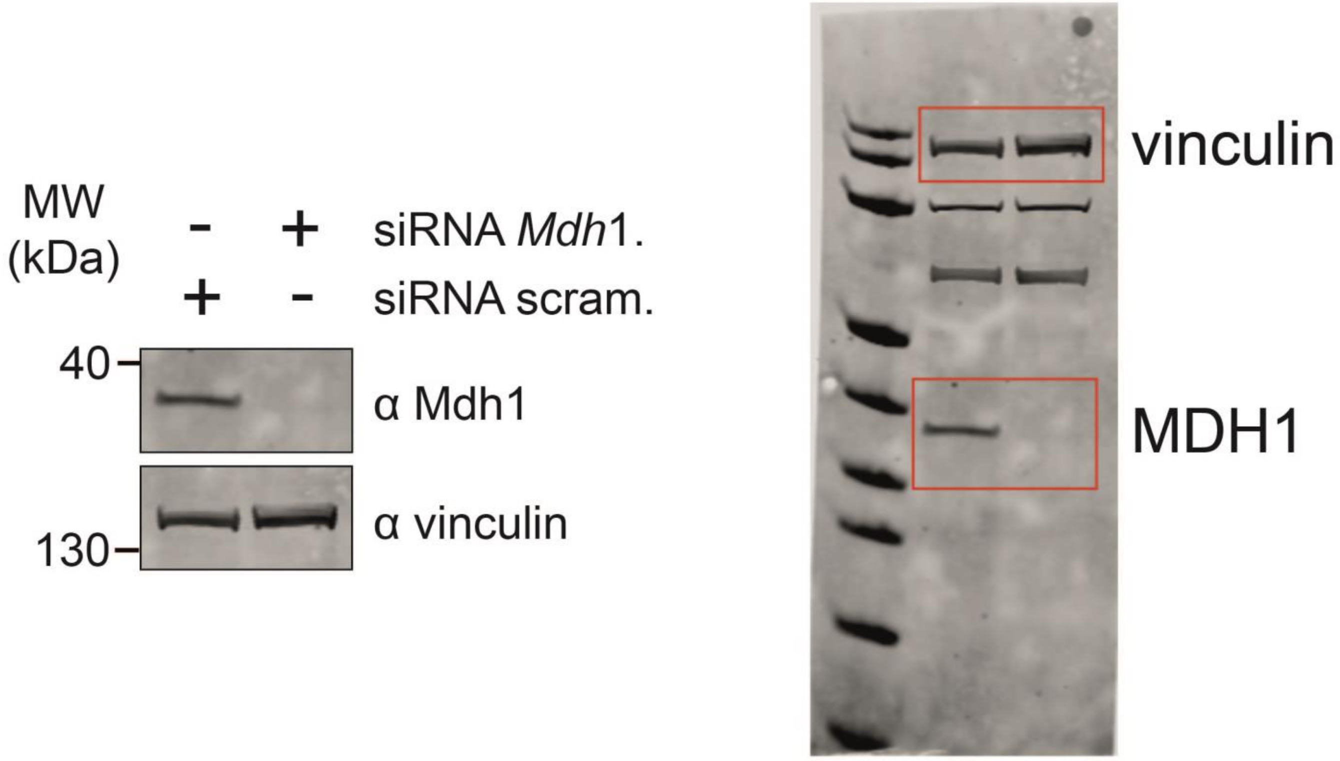

Mahmood et al - Extended Data Figure 2K

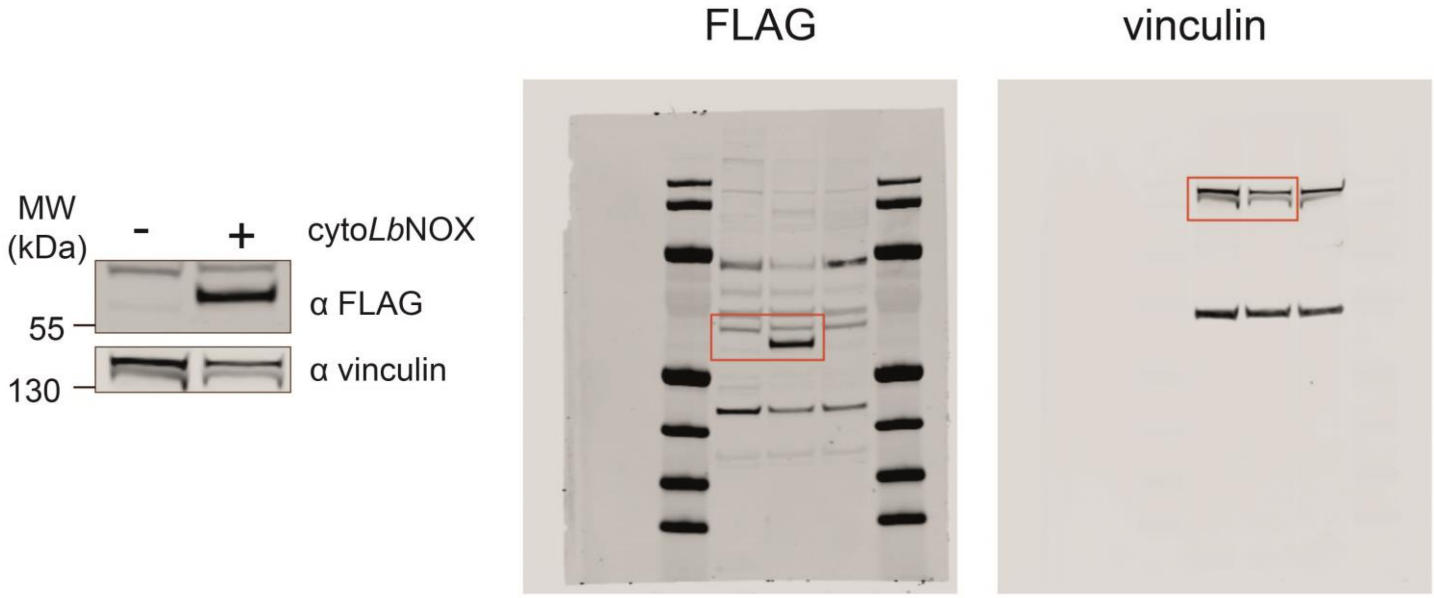

Mahmood et al - Extended Data Figure 2Q

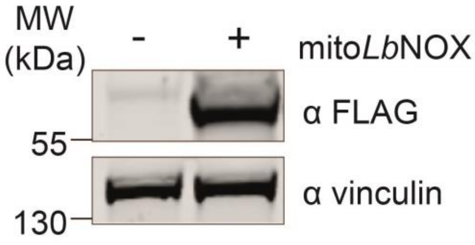

FLAG

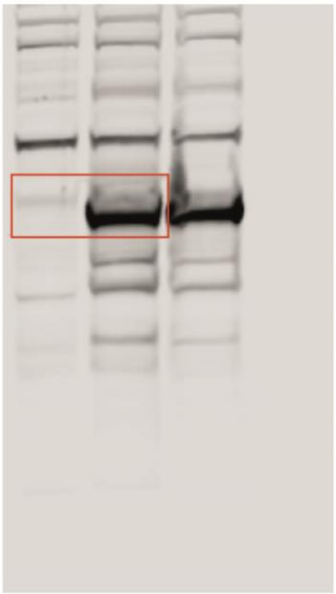

vinculin

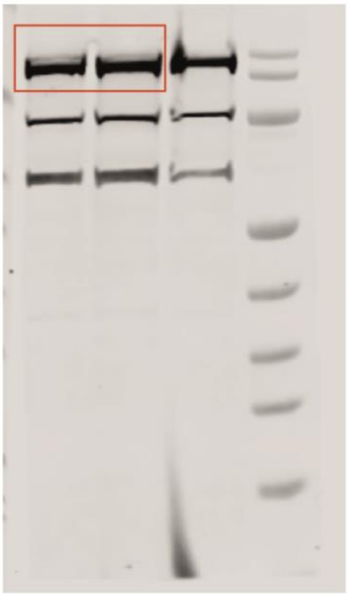

Mahmood et al - Extended Data Figure 3A-C

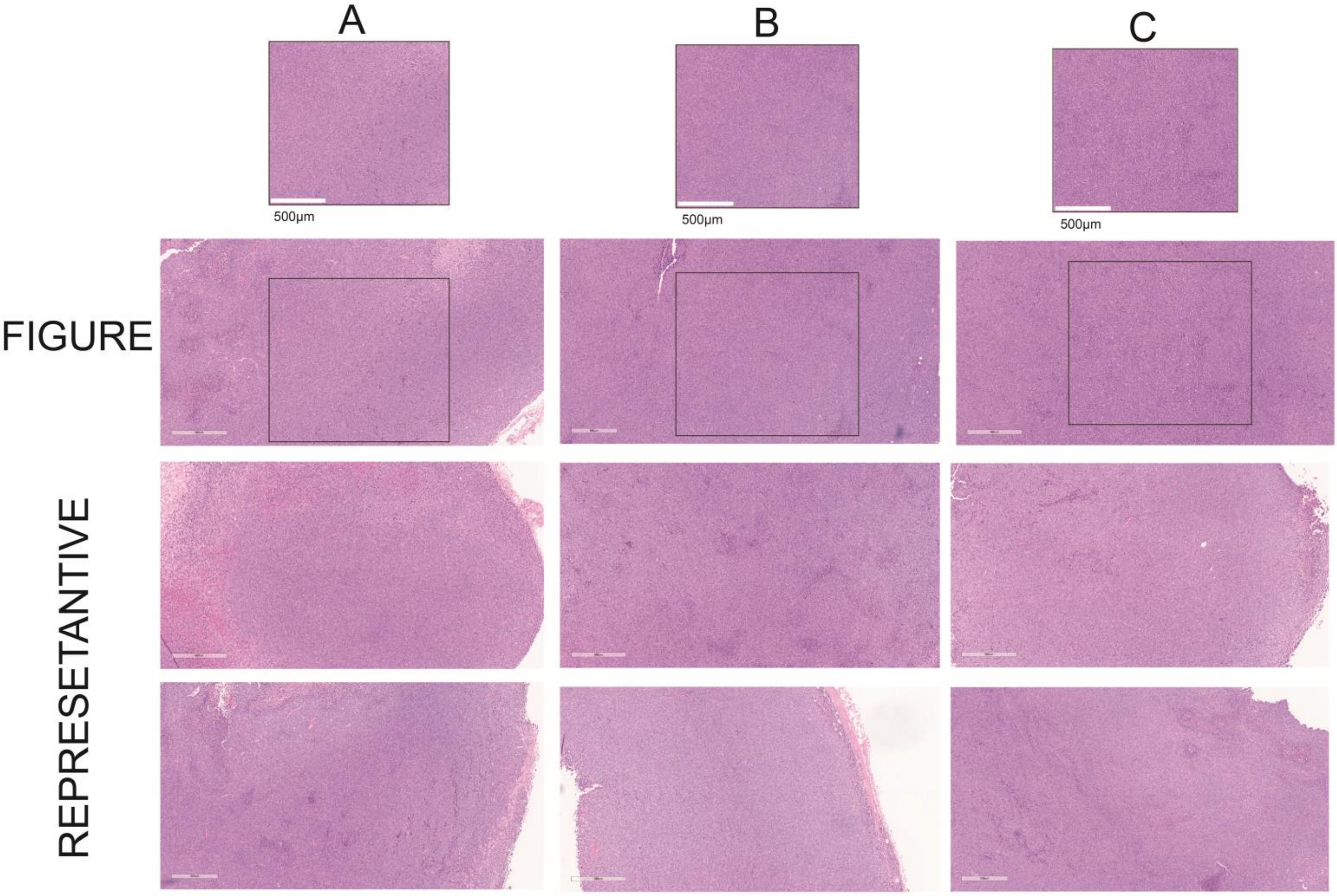

Mahmood et al - Extended Data Figure 5B

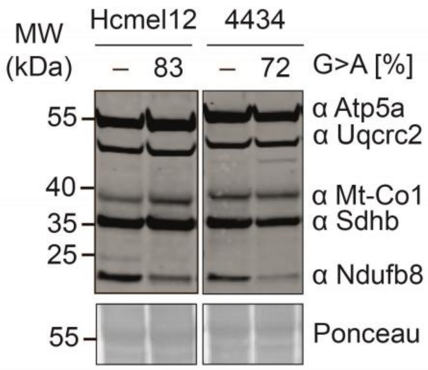

HCMEL12 BLOT

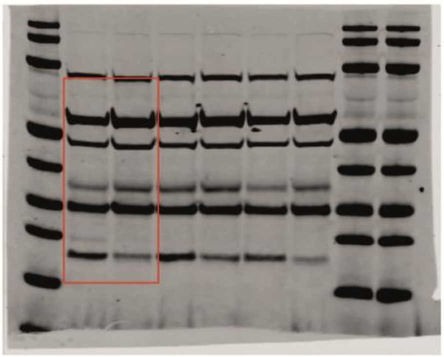

HCMEL12 PONCEAU

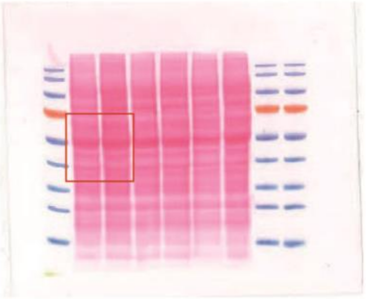

4434 BLOT

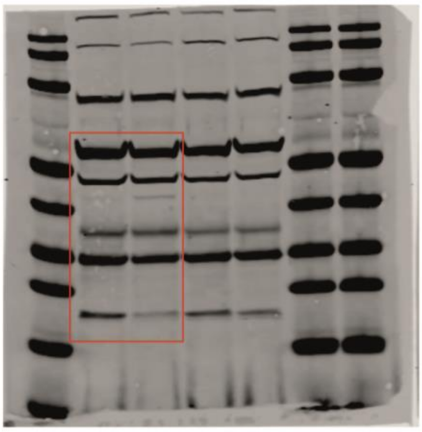

4434 PONCEAU

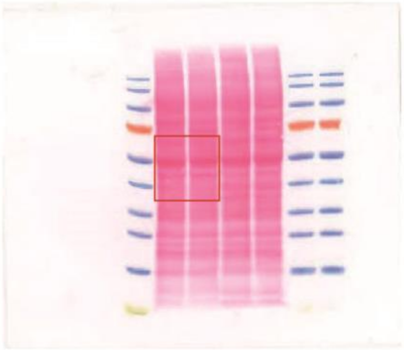

Mahmood et al - Extended Data Figure 7A-B

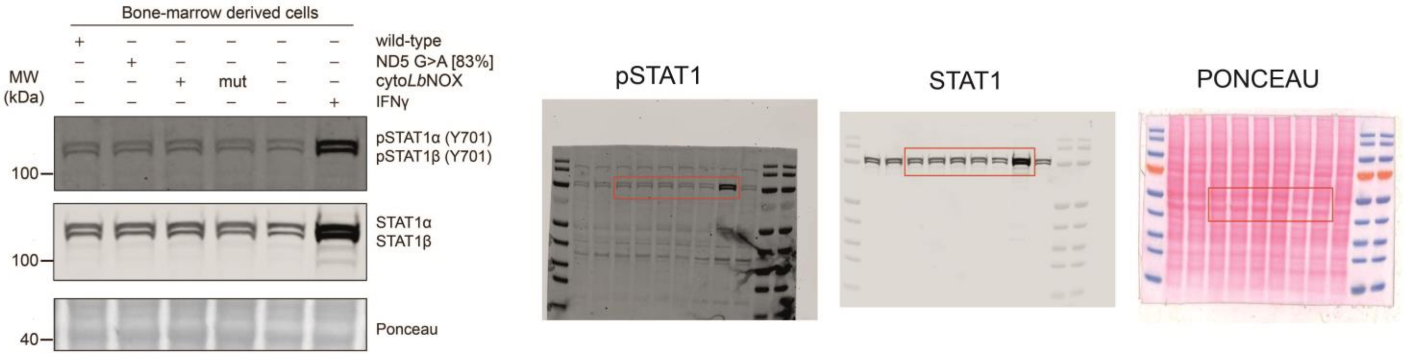

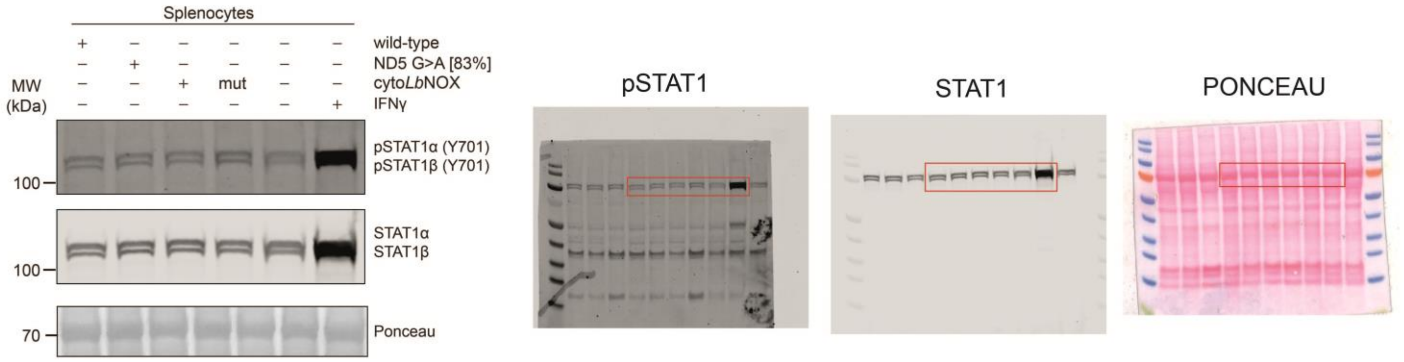

Mahmood et al - Extended Data Figure 7B + D

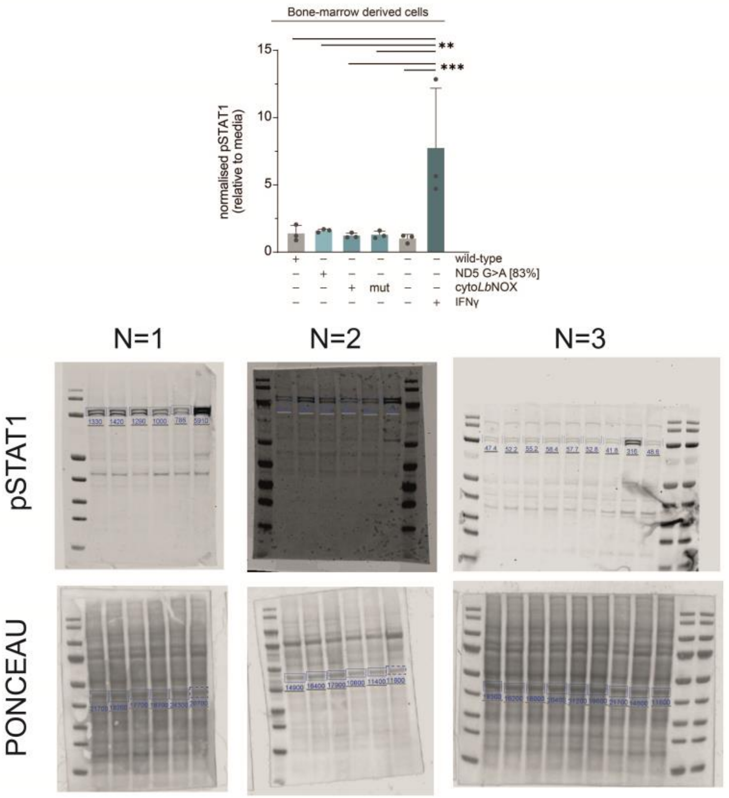

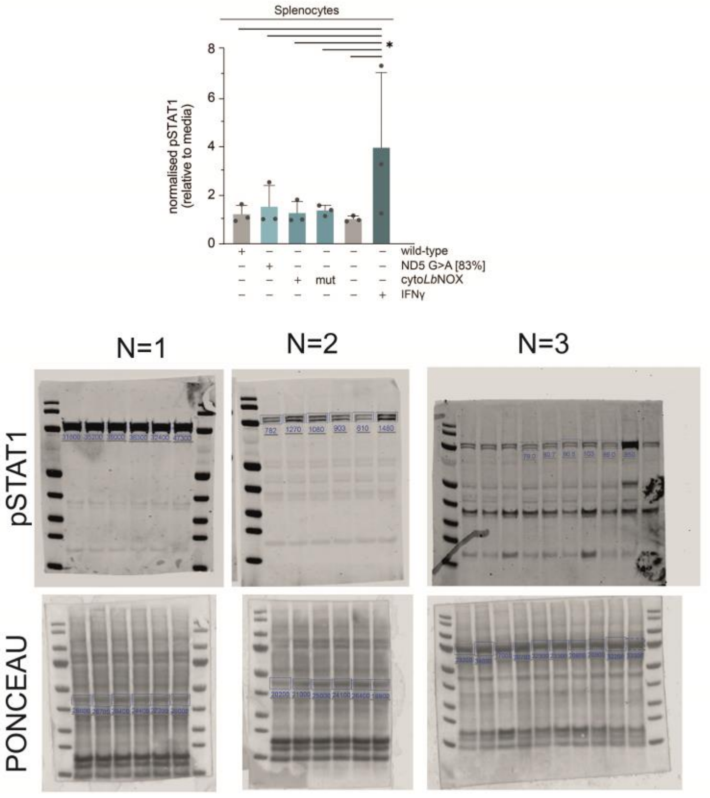

Mahmood et al - Extended Data Figure 9A-B

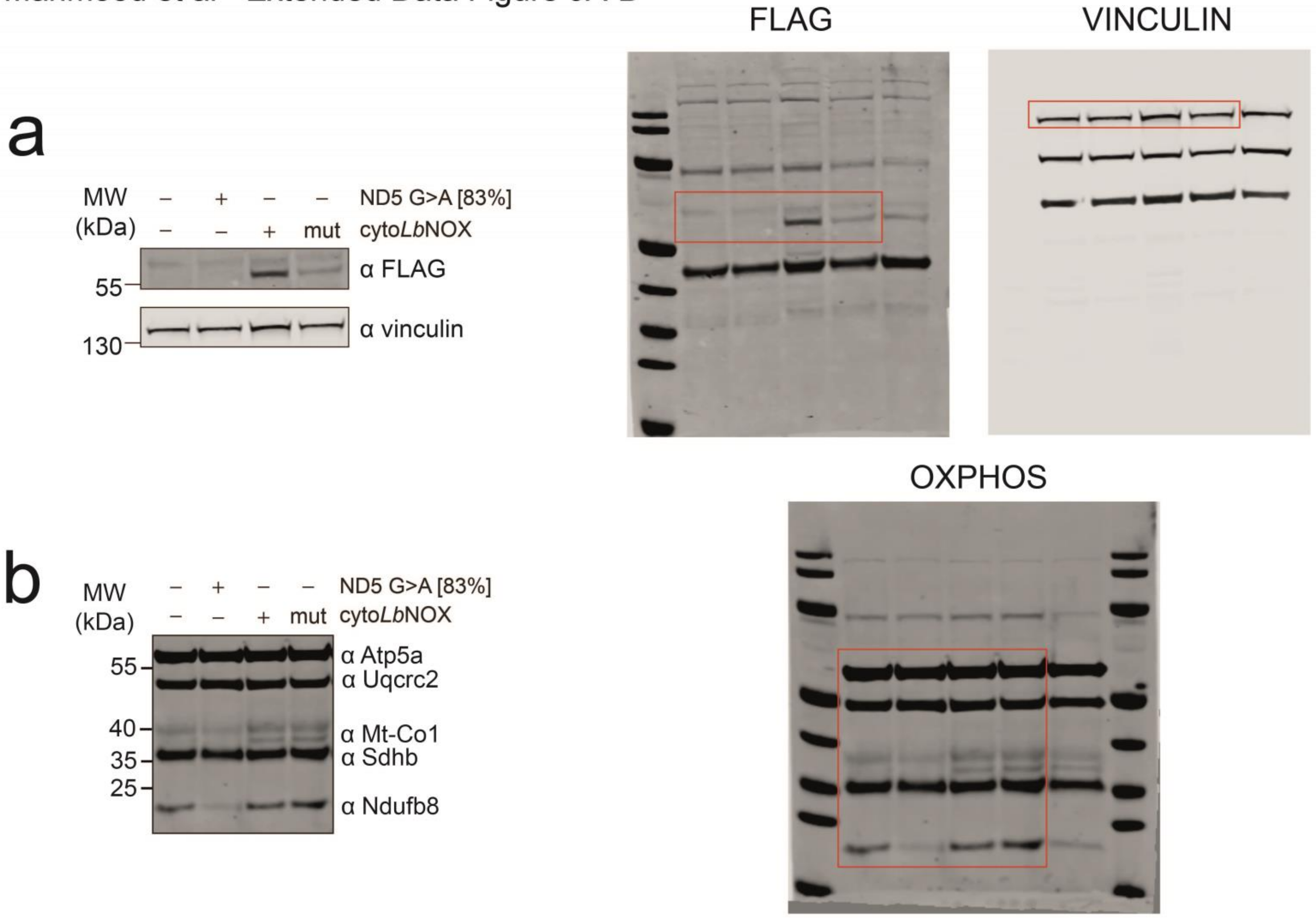

Supplement: Supplementary file 3 — Source Data Fig. 1 Unprocessed western Blots and/or gels. Source Data Fig. 4 Unprocessed tumor images. Source Data Extended Data Fig. 1 Unprocessed western Blots and/or gels. Source Data Extended Data Fig. 2 Unprocessed western Blots and/or gels. Source Data Extended Data Fig. 3 Unprocessed tumor H&E stains. Source Data Extended Data Fig. 5 Unprocessed western Blots and/or gels. Source Data Extended Data Fig. 7 Unprocessed western Blots and/or gels. Source Data Extended Data Fig. 9 Unprocessed western Blots and/or gels. [file 43018_2023_721_MOESM3_ESM.pdf]
